# Initial community composition determines the long-term dynamics of a microbial cross-feeding interaction by modulating niche availability

**DOI:** 10.1038/s43705-022-00160-1

**Published:** 2022-08-24

**Authors:** Jan Dolinšek, Josep Ramoneda, David R. Johnson

**Affiliations:** 1 Department of Environmental Microbiology, Swiss Federal Institute of Aquatic Science and Technology (Eawag), 8600 Dübendorf, Switzerland; 2 Department of Environmental Systems Science, Swiss Federal Institute of Technology (ETH), 8092 Zürich, Switzerland; 3 Cooperative Institute for Research in Environmental Sciences, University of Colorado, Boulder, CO USA

**Keywords:** Microbial ecology, Microbial ecology

## Abstract

Multi-step substrate consumption pathways can promote microbial biodiversity via cross-feeding. If one cell type preferentially consumes a primary substrate rather than the subsequently formed intermediates, then other cell types can specialize in consuming the intermediates. While this mechanism for promoting biodiversity is established, predicting the long-term persistence of such cross-feeding interactions remains challenging. Under what conditions will the interaction (and thus biodiversity) persist or disappear? To address this question, we propagated co-cultures of two isogenic strains of the bacterium *Pseudomonas stutzeri*. One completely reduces nitrate to nitrogen gas but preferentially reduces nitrate rather than nitrite (referred to as the generalist), while the other only reduces nitrite to nitrogen gas (referred to as the specialist). We found that the two strains coexist via nitrite cross-feeding when grown together, but the initial ratio of specialist-to-generalist (r_S/G_) determines the long-term dynamics of the co-culture. Co-cultures with large initial r_S/G_s converge to the same r_S/G_ and persist thereafter. Co-cultures with small initial r_S/G_s also converge to the same r_S/G_ but then become increasingly dominated by the generalist. The likely cause of these different dynamics is that the initial r_S/G_ determines the initial environment, which in turn determines the initial selection pressures and phenotypes acquired by the generalist. Our results demonstrate that initial community composition controls the long-term dynamics and persistence of a cross-feeding interaction, and is therefore an important factor for community development and for engineering communities to achieve desired outcomes.

## Introduction

**Fig. 1:**
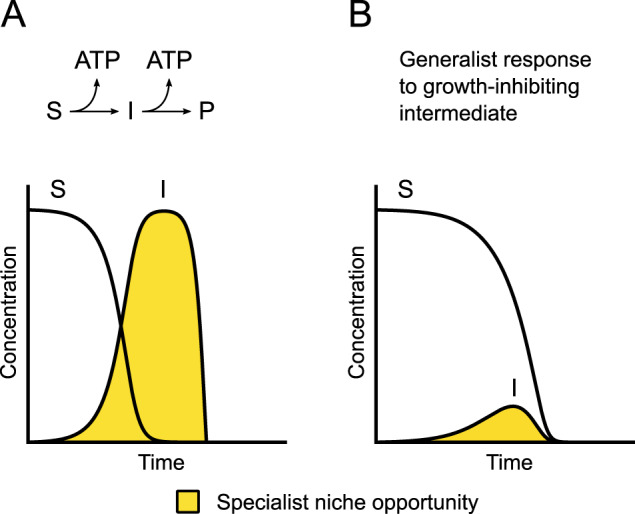
Intermediate accumulation during operation of multi-step substrate consumption pathways. Consider a multi-step substrate consumption pathway where a substrate (S) is consumed via an intermediate (I) into an end product (P). **A** If a generalist performs the entire pathway but preferentially consumes S rather than I, then I will accumulate (yellow shaded region). This creates a niche for the specialist, where the specialist consumes I, and can enable the coexistence of the generalist and specialist via a cross-feeding interaction. **B** If phenotypic changes that improve the consumption of I are more readily available or beneficial to the generalist than the specialist, then this would reduce the duration of the niche for the specialist (yellow shaded region) and potentially disrupt the cross-feeding interaction.

Multi-step substrate consumption pathways consist of a series of enzymatic reactions where a primary substrate is consumed via one or more intermediates into an end product (Fig. 1A). Their operation often results in the extracellular accumulation of intermediates (Fig. 1A; yellow shaded region) [1–5]. For example, if a cell preferentially consumes a primary substrate rather than the subsequently formed intermediates, then the intermediates will accumulate within the cell. They may then leak or be transported out of the cell, resulting in their extracellular accumulation [1–5]. After the primary substrate is sufficiently depleted, the cell may then take up and consume the intermediates [1–5]. A canonical example is overflow metabolism, where cells incompletely oxidize substrates to metabolites that could, in principle, be further oxidized to generate additional ATP [2, 3, 5].

The extracellular accumulation of intermediates could be an important mechanism that promotes biodiversity within microbial communities [6–10]. Consider a cell type that completely consumes a primary substrate via an intermediate into an end product (referred to hereafter as the generalist) (Fig. 1A). If the intermediate accumulates outside the cell to growth-supporting concentrations, then this creates a niche for another cell type to occupy (Fig. 1A; yellow shaded region), where the other cell type specializes at consuming the intermediate (referred to hereafter as the specialist). The generalist and specialist could then coexist via a cross-feeding interaction. Indeed, evolution experiments with the bacterium *Escherichia coli* repeatedly observed the emergence of generalists and specialists that coexist via this type of cross-feeding [11–13].

While the extracellular accumulation of intermediates can promote biodiversity via cross-feeding [6–10], the long-term fate of the interaction after its origin is less clear. Under what conditions is the cross-feeding interaction likely to persist or disappear? Either partner could acquire new phenotypes via genetic, epigenetic, or regulatory changes that reinforce or disrupt the interaction [9, 14, 15]. We postulate here some possible outcomes of phenotypic change that are linked to the duration of the niche created by the generalist for the specialist (i.e., the length of time during which the intermediate is available [Fig. 1; yellow shaded regions]). If the intermediate accumulates to growth-inhibiting concentrations (e.g., direct or indirect toxicity, thermodynamic constraints, etc.), then the generalist may acquire phenotypes that improve the consumption of the intermediate or reduce the consumption of the primary substrate, both of which would reduce the intermediate accumulation (Fig. 1B). This would deprive the specialist of the intermediate (i.e., reduce the duration of the niche [Fig. 1B; yellow shaded region]) and potentially disrupt the cross-feeding interaction and deteriorate biodiversity. Alternatively, if phenotypic changes that improve the consumption of the intermediate are more readily accessible or beneficial to the specialist than to the generalist, then the specialist would have long-term access to the intermediate. This would promote the persistence of the cross-feeding interaction and biodiversity.

Our objectives here were two-fold. First, we experimentally tested whether a specialist can indeed coexist with a generalist via a cross-feeding interaction, where the generalist preferentially consumes a primary substrate into a growth-supporting intermediate while the specialist consumes the intermediate. We therefore sought to experimentally recreate the origin of a cross-feeding interaction. Importantly, we postulated that a single mutation is sufficient to create a cross-feeding interaction. Second, after its origin, we sought to evaluate the long-term dynamics of the cross-feeding interaction and link the dynamics to phenotypic changes in intermediate production and consumption.

**Fig. 2:**
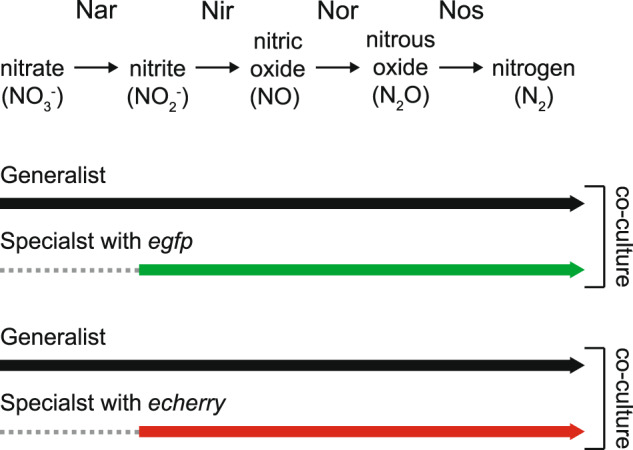
Experimental system used in this study. Our system consists of three isogenic strains of *P. stutzeri*. The generalist has the complete denitrification pathway and can reduce nitrate to nitrogen gas. The two specialists have a single loss-of-function mutation in the *narG* gene and can reduce nitrite to nitrogen gas but cannot reduce nitrate to nitrite. The two specialists are genetically identical except that they have a chromosomally located green (*egfp*) or red (*echerry*) fluorescent protein-encoding gene. When the generalist is grown together with one of the two specialists under anoxic conditions and with nitrate as the growth-limiting substrate, the generalist preferentially consumes nitrate to nitrite while the specialist consumes the released nitrite. Thick arrows indicate the nitrogen oxides that can be consumed by each strain. Nar nitrate reductase, Nir nitrite reductase, Nor nitric oxide reductase, Nos nitrous oxide reductase.

To achieve these objectives, we used experimental co-cultures consisting of two isogenic strains of the denitrifying bacterium *Pseudomonas stutzeri* A1501 that differ at a single genetic locus (Fig. 2) [16], thus minimizing confounding factors that can emerge when using more distantly related microorganisms. One strain sequentially reduces nitrate (NO_3_^-^) to nitrite (NO_2_^-^), nitric oxide (NO), nitrous oxide (N_2_O), and finally to nitrogen gas (N_2_) (referred to as the generalist) (Fig. 2) [16]. When the generalist is grown alone in an anoxic environment with nitrate as the growth-limiting resource, it accumulates nitrite outside the cell and subsequently reduces nitrite only after nitrate is sufficiently depleted [16]. The hypothesized mechanism for this is that the nitrate reductase has a higher affinity for intracellular electron carriers than the nitrite reductase, and thus has a higher activity when nitrate is present [16]. The main point of this study is that the generalist accumulates extracellular nitrite and exhibits dynamics qualitatively similar to those in Fig. 1A. The other strain has a single loss-of-function deletion in the nitrate reductase-encoding *narG* gene (referred to hereafter as the specialist) (Fig. 2) and reduces nitrite to nitrogen gas but cannot reduce nitrate to nitrite [16]. Using these two strains, we first assembled the generalist and specialist together into co-cultures and tested whether they can coexist via a nitrite cross-feeding interaction. We next serially transferred the co-cultures, evaluated the long-term dynamics of the nitrite cross-feeding interaction after its origin, and quantified phenotypic changes to nitrite production and consumption.

## Materials and methods

### Strains and growth media

We used two isogenic strains of *P. stutzeri* A1501 [17] for all our experiments (Fig. 2 and Supplementary Table S1), both of which are described in detail elsewhere [16, 18, 19]. The generalist has a complete denitrification pathway while the specialist has a single loss-of-function deletion in the nitrate reductase-encoding *narG* gene that prevents growth with nitrate under anoxic conditions (Fig. 2 and Supplementary Table S1) [16]. Both strains have an intact periplasmic nitrate reductase (encoded by the *nap* genes), but it does not support growth under anoxic conditions [16, 20]. The specialist additionally has a chromosomally located isopropyl-β-D-thiogalactopyranoside (IPTG)-inducible fluorescent protein-encoding gene (*egfp* or *echerry* [21]) (Fig. 2 and Supplementary Table S1) [18], which allows us to quantify the frequencies of each strain when grown together [22]. All strains have a chromosomally located gentamycin resistance-encoding gene that enables us to grow the strains in the presence of gentamycin and minimize the probability of culture contamination (Supplementary Table S1) [22]. All strains also have a loss-of-function deletion in the *comA* gene (Supplementary Table S1) [16], which prevents the uptake of extracellular DNA and minimizes the probability that the strains will recombine with each other when grown together [23]. We provided a complete description of strain construction elsewhere [16, 18, 19]. Briefly, we deleted the *narG* and *comA* genes using derivatives of the pAW19 plasmid [16, 24] and introduced the gentamycin resistance- and fluorescent protein-encoding genes using mini-Tn7T transposons [18, 19, 25].

We grew each strain alone under oxic conditions using lysogeny broth (LB) medium or a completely defined synthetic citrate-asparagine medium (ACS medium) [26] supplemented with 10 mg/L gentamycin. We confirmed that the strains have identical growth properties in oxic ACS medium without IPTG; neither deletion of the *narG* gene nor the insertion of the fluorescent protein-encoding genes had experimentally observable effects on growth (Supplementary Fig. S1). We grew the strains alone or together in anoxic ACS medium purged with nitrogen gas and amended with sodium nitrate as the growth-limiting substrate (concentrations for individual experiments are reported in the results section), 10 mg/L gentamycin, and 1 mM sodium bromide. We amended bromide to the medium to serve as an internal reference for ion chromatography (IC) measurements of nitrogen oxides as described elsewhere [27]. However, we found that chloride was more effective as an internal reference (see the Supplementary Information). Finally, we grew the strains together in a medium with the pH set to 6.5 or 7.5 as described elsewhere [16]. Nitrite is conditionally toxic depending on pH, where it has no experimentally observable effects on growth at pH 7.5 (weak nitrite toxicity) but severe deleterious effects at pH 6.5 (strong nitrite toxicity) [16]. This allows us to experimentally manipulate nitrite toxicity and measure the consequences [16, 18, 22, 28]. We note that pH itself has no quantifiable effects on growth under our experimental conditions across this pH range [16].

### Duration of nitrite availability experiment

We quantified the extent of time during which nitrite is available for consumption (referred to hereafter as the “duration of nitrite availability”). This quantity measures the duration of the niche created by the generalist for the specialist. To achieve this, we first inoculated the generalist into oxic ACS medium set to pH 6.5 (strong nitrite toxicity) or 7.5 (weak nitrite toxicity) and amended with 10 mg/L gentamycin, and incubated the cultures overnight. After reaching the stationary phase, we diluted the cultures 100-fold into 200 µL fresh ACS medium amended with 0.9, 1.6, 2.6, 4.3, 7.2, 12, or 20 mM nitrate and 10 mg/L gentamycin in a 96-well microtiter plate. We then transferred the plate into an anoxic glove box (Coy Laboratory Products, Grass Lake, MI) containing a nitrogen:hydrogen (97:3) atmosphere and allowed the plate to equilibrate with the anoxic atmosphere for 15–20 min. We finally sealed the plate with Topseal-A PLUS transparent film (Perkin Elmer, Schwerzenbach, Switzerland) and incubated it in an Epoch2 plate reader (Biotek, Luzern, Switzerland) located inside the glove box. We used the following settings for the plate reader: temperature, 34 °C; shaking, continuous; optical density at 600 nm (OD_600_), every 6 min; total incubation time, 72 h.

We quantified the duration of nitrite availability using the OD_600_-based cell density measurements that we collected over time. For the ancestral generalist, we quantified the duration as the time difference between the first peak in the growth rate (corresponding to the maximum rate of nitrite production) and the time at which growth with nitrite reached its maximum (corresponding to the maximum rate of nitrite consumption). For the evolved generalists, we quantified the duration as the time difference between the time the OD_600_ exceeded 0.06 (typically between 25% of the maximal OD_600_) and the time at which growth with nitrite reached its maximum (corresponding to the maximum rate of nitrite consumption).

To quantify the duration of nitrite availability, we first smoothed the OD_600_ data to minimize the impact of potential outlier measurements by applying a moving average across ten time-consecutive measurements. We then removed the background signal by subtracting the lowest OD_600_ value from each dataset. We finally quantified the time difference between the two timepoints specified above. We verified that the durations of nitrite availability measured from the OD_600_-based cell density data are accurate proxy measures of the true durations of nitrite availability using bioreactor assays (Supplementary Fig. S2). Briefly, we operated bioreactors with different pH or initial nitrate concentrations and quantified cell density with flow cytometry (FC) as described elsewhere [29, 30] and nitrate and nitrite concentrations with IC as described in Supplementary Information.

### Initial ratio experiments

We tested whether the generalist and specialist can coexist via a nitrite cross-feeding interaction using initial ratio experiments. To achieve this, we inoculated each strain alone into oxic ACS medium set to pH 6.5 (strong nitrite toxicity) or 7.5 (weak nitrite toxicity) and amended with 10 mg/L gentamycin, and incubated the cultures overnight as described for the duration of nitrite availability experiment. After reaching the stationary phase, we mixed the generalist and specialist together at different initial ratios of specialist-to-generalist (r_S/G_). One set of these co-cultures consisted of the specialist with *egfp* while another consisted of the specialist with *echerr*y (Fig. 2). We used both specialists to periodically monitor for cross-contamination between the co-cultures, which we never observed. We next diluted the co-cultures 100-fold into 200 µL fresh ACS medium set to pH 6.5 or 7.5 and amended with 10 mM nitrate and 10 mg/L gentamycin in 96-well microtiter plates. We then moved the plates into the anoxic glove box, incubated the co-cultures under anoxic conditions, and quantified OD_600_ over time as described for the duration of the nitrite availability experiment.

After incubation of the microtiter plates, we serially transferred the co-cultures. Briefly, at the end of the 72 h-incubation time, we removed the microtiter plate from the plate reader while retaining the plate inside the anoxic glove box, removed the transparent film from the microtiter plate, and diluted each co-culture 100-fold into 200 µL fresh anoxic ACS medium set to pH 6.5 (strong nitrite toxicity) or 7.5 (weak nitrite toxicity) and amended with 10 mM nitrate and 10 mg/L gentamycin in a new 96-well microtiter plate. We then sealed the new plate with transparent film and incubated it under anoxic conditions as described above. We repeated this procedure 12 times for co-cultures serially transferred at pH 6.5 and seven times for co-cultures serially transferred at pH 7.5. Because the co-cultures entered the stationary phase before each transfer, the total number of generations during the experiment was approximately 80 at pH 6.5 and 47 at pH 7.5. These estimates are based on 6.644 generations from a 100-fold dilution (i.e., 2^6.644^ = 100). Throughout the experiment, we quantified the r_S/G_ for each co-culture using a spiral plating method in conjunction with fluorescence photography as described in the Supplementary Information and Supplementary Fig. S3.

At the end of the initial ratio experiment, we streaked aliquots from co-cultures serially transferred at pH 6.5 (strong nitrite toxicity) along with their respective ancestral control cultures onto LB agar plates, incubated them at 30 °C, and randomly selected 70 isolated generalist colonies from each of six co-cultures (total of 420 isolates) initiated with different r_S/G_s and from four ancestral control cultures (additional 280 isolates). We then grew the generalist isolates alone with oxic LB medium amended with 10 mg/L gentamycin in microtiter plates. After reaching the stationary phase, we removed a 100-µl aliquot from each generalist isolate culture and deposited it into 100 µl of fresh oxic LB medium amended with 30% glycerol (vol:vol) and 10 mg/L gentamycin. We finally vortexed the mixtures and archived the isolates at –80 °C.

### Phenotypic properties of generalist isolates

Using the archived generalist isolates from the six co-cultures and four ancestral control cultures serially transferred at pH 6.5 (strong nitrite toxicity), we measured the phenotypic properties of each generalist isolate. We transferred an aliquot of each archived isolate into fresh oxic LB medium amended with 10 mg/L gentamycin using a 96-pin plate replicator. After overnight growth, we transferred the generalist isolate cultures into 200 µL of fresh ACS medium set to pH 6.5 (strong nitrite toxicity) and amended with 10 mM nitrate and 10 mg/L gentamycin in 96-well microtiter plates using a 96-pin plate replicator. We then moved the microtiter plates into the anoxic glove box, incubated the generalist isolates under anoxic conditions, and quantified OD_600_ over time as described for the duration of the nitrite availability experiment. For each generalist isolate, we used the OD_600_ measurements to quantify the maximum cell density (maximum observed OD_600_), the duration of nitrite availability, and the maximum observed growth rates with nitrate and nitrite. To quantify the maximum observed growth rates, we log-transformed the smoothed and background corrected OD_600_ measurements, calculated the slope for all possible sets of five time-consecutive measurements, and identified the maximum slope.

### Genome analyses

We sequenced the genomes of the generalist isolates serially transferred at pH 6.5 (strong nitrite toxicity) to test whether there are putative genetic changes between the isolates and their respective ancestors. The procedures are described in detail in the Supplementary Information and follow established protocols [31–33]. We reported all putative genetic changes in Supplementary Table S2 and deposited all the raw sequence reads in the NCBI Sequence Read Archive (https://www.ncbi.nlm.nih.gov/sra) under BioSample accessions SAMN20157770–SAMN20157812.

### Mathematical models and simulations

We used adapted versions of dynamical models formulated and described in detail elsewhere [16, 34]. The model simulates primary substrate consumption and intermediate production by multiple cell types growing within the same completely mixed physical compartment [16, 34]. The growth of individual cell types is described by the Monod function. To simulate the initial ratio experiments, we used the cell-type composition at the end of each simulation cycle to initiate the next simulation cycle, corrected for the 100-fold dilution factor. We provide a complete description of the mathematical model and model parameters in the Supplementary Information.

### Statistics

We used two-sided two-sample *t*-tests for pairwise comparisons, two-sided Pearson correlation tests for associations between two variables, and Mann–Kendall trend tests for associations between individual variables and time. We adjusted *p* values for multiple comparisons using the Holm–Bonferroni method. We reported the test type, *p* value (*p*), and sample size (*n*) for each test in the results section. All sample sizes refer to the number of biological replicates (for pairwise comparisons and correlation tests) or timepoints (trend tests). We considered *p* < 0.05 to be statistically significant.

## Results

### The generalist accumulates extracellular nitrite

We first tested whether the generalist accumulates substantial extracellular nitrite under our experimental conditions, and thus creates a niche for the specialist. To accomplish this, we grew the generalist alone in bioreactors with anoxic ACS medium amended with 12 mM nitrate as the growth-limiting substrate and measured the extracellular concentrations of nitrate and nitrite over time. We performed these experiments at pH 6.5 (strong nitrite toxicity) and 7.5 (weak nitrite toxicity).

**Fig. 3:**
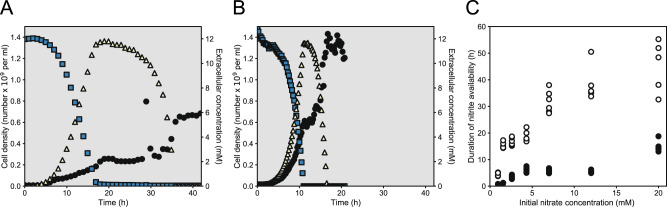
Growth and nitrogen oxide dynamics of the generalist in batch culture. We grew the generalist alone in a bioreactor at **A** pH 7.5 (weak nitrite toxicity) or **B** pH 6.5 (strong nitrite toxicity) under anoxic conditions with nitrate as the growth-limiting substrate. Blue squares are measured extracellular nitrate concentrations, yellow triangles are measured extracellular nitrite concentrations, and black circles are measured cell densities. We measured extracellular nitrate and nitrite concentrations with IC and cell densities with FC. **C** Measured durations of nitrite availability for the generalist growing in batch culture. We grew the generalist alone in 96-well microtiter plates under anoxic conditions with nitrate as the growth-limiting substrate. Open symbols are durations of nitrite availability at pH 6.5 and closed symbols are durations of nitrite availability at pH 7.5. Each symbol is an independent biological replicate.

We observed a substantial accumulation of extracellular nitrite regardless of the pH (Fig. 3A, B). When grown at pH 6.5 (strong nitrite toxicity), extracellular nitrite accumulated to a concentration comparable to the initial nitrate concentration (measured maximum extracellular nitrite concentration, 11.8 mM; measured initial nitrate concentration, 12.0 mM) and was subsequently consumed to below the detection limit (Fig. 3A). When grown at pH 7.5 (weak nitrite toxicity), extracellular nitrite again accumulated to a concentration comparable to the initial nitrate concentration (measured maximum extracellular nitrite concentration, 11.7 mM; measured initial nitrate concentration, 12.9 mM) and was subsequently consumed to below the detection limit (Fig. 3B). During growth at pH 6.5, substantial nitrite consumption did not begin until a prolonged period of time after nitrate consumption was complete, resulting in a relatively long duration of nitrite availability (Fig. 3A). During growth at pH 7.5, in contrast, substantial nitrite consumption began immediately after nitrate consumption was complete, resulting in a relatively short duration of nitrite availability (Fig. 3B). The longer duration of nitrite availability at pH 6.5 indicates that the duration of the niche created by the generalist for the specialist depends on pH.

To routinely quantify the duration of nitrite availability, we grew the generalist alone with varying amounts of nitrate as the growth-limiting substrate. We then quantified the length of time from when the growth rate with nitrate was maximum to when the growth rate with nitrite was maximum. This cell density-based proxy measure is valid because the growth of the generalist is directly linked to the consumption of nitrate and nitrite (Fig. 3A, B). The cell density of the generalist was initially linearly correlated with nitrate consumption at both pH 6.5 (strong nitrite toxicity) (two-sided Pearson correlation test; *r* = −0.96, *p* = 1.5 × 10^–8^, *n* = 15) (Fig. 3A) and 7.5 (weak nitrite toxicity) (two-sided Pearson correlation test; *r* = −1.00, *p* = 2.2 × 10^–16^, *n* = 30) (Fig. 3B). After nitrate was depleted, the cell density of the generalist became linearly correlated with nitrite consumption at both pH 6.5 (strong nitrite toxicity) (two-sided Pearson correlation test; *r* = −0.97, *p* = 3 × 10^–4^, *n* = 7) (Fig. 3A) and 7.5 (weak nitrite toxicity) (two-sided Pearson correlation test; *r* = −0.97, *p* = 6.8 × 10^–10^, *n* = 16) (Fig. 3B). We further validated our cell density-based approach by testing for concordance with our IC-based direct measures of the duration of nitrite availability. We observed a significant positive and linear relationship between the cell density- and IC-based measures (two-sided Pearson correlation test; *r* = 0.999, *p* = 0.023, *n* = 3) (linear regression model; slope = 1.19, intercept = −2.31, *r*^2^ = 0.99) (Supplementary Fig. S2), which further validates our cell density-based approach to routinely estimate the duration of nitrite availability.

Using our cell density-based approach, we found that the duration of nitrite availability was significantly longer at pH 6.5 (strong nitrite toxicity) than at 7.5 (weak nitrite toxicity) regardless of the initial nitrate concentration (two-sample two-sided *t*-tests; Holm-adjusted *p* < 1.7 ×; 10^–3^, *n* = 5) (Fig. 3C). Moreover, at both pH 6.5 and 7.5, the duration of nitrite availability increased linearly with the initial concentration of nitrate (two-sided Pearson correlation tests; *r* > 0.92, Holm-adjusted *p* < 0.005, *n* = 7) (Fig. 3C). Taken together, our results indicate that the generalist does indeed accumulate substantial amounts of extracellular nitrite (Fig. 3A, B), thus creating a potential niche for the specialist, and that the duration depends on the initial nitrate concentration and pH (Fig. 3C). We note that the underlying mechanism causing the longer duration at pH 6.5 is unclear, but is likely linked to the increased toxicity of nitrite at this pH [16]. Regardless, the relevant point here is that we can use the pH to experimentally manipulate the duration of nitrite availability and measure the consequences on the growth of the specialist.

### Origin of a nitrite cross-feeding interaction

We next tested whether the extracellular accumulation of nitrite by the generalist enables the origin of a nitrite cross-feeding interaction with the specialist. We used coexistence as a strict condition for evaluating whether we could successfully recreate the origin. To test for coexistence, we performed reciprocal initial ratio experiments, which provide definitive empirical evidence of coexistence [35]. Briefly, if the generalist and specialist can each increase in frequency from rare, then they can coexist. We tested this by serially transferring co-cultures of the generalist and specialist initiated at different ratios of specialist-to-generalist (r_S/G_). We then quantified changes in the log r_S/G_ over the first three co-culture transfers, which is the fewest number of transfers that still allow us to perform statistical trend testing (*n* = 4 for the Mann–Kendall trend test). We used the fewest number of transfers to minimize the probability that genetic and phenotypic changes would emerge over the timecourse of the experiment.

**Fig. 4:**
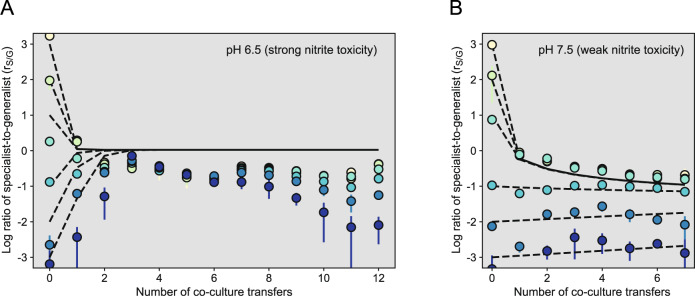
Dynamics of r_S/G_ during serial batch transfer. We mixed the generalist and specialist together at different initial r_S/G_s and grew them at **A** pH 6.5 (strong nitrite toxicity) or **B** pH 7.5 (weak nitrite toxicity) under anoxic conditions with nitrate as the growth-limiting substrate. We incubated the co-cultures until reaching the stationary phase and then diluted them 1:100 (vol:vol) into a fresh medium (referred to as a transfer). We measured the r_S/G_s using a spiral plating method in conjunction with fluorescence photography immediately before each subsequent transfer. Data points are means and error bars are one standard error of two replicate experiments. Dashed lines are model simulations for co-cultures initiated at the same r_S/G_s used for the experiments.

We first tested whether the generalist can increase in frequency from rare. To achieve this, we used large initial log r_S/G_s (mean measured initial log r_S/G_s of 3.23 and 1.98 at pH 6.5 [strong nitrite toxicity] and 2.98 and 2.12 at pH 7.5 [weak nitrite toxicity]). After the co-cultures reached the stationary phase, we measured the final log r_S/G_s and transferred the co-cultures into a fresh medium. At both pH 6.5 and 7.5, we found that the log r_S/G_s decreased over the first three transfers (Mann–Kendall trend test; tau = −1*, p* = 0.042) (Fig. 4). Thus, the generalist can indeed increase in frequency from rare regardless of nitrite toxicity.

We next tested whether the specialist can also increase in frequency from rare. To achieve this, we used small initial log r_S/G_s (mean measured initial log r_S/G_s of −3.19 and −2.65 at pH 6.5 [strong nitrite toxicity] and −3.33 and −2.13 at pH 7.5 [weak nitrite toxicity]). At pH 6.5 (strong nitrite toxicity), we found that the log r_S/G_s increased over the first three transfers (Mann–Kendall trend test; tau = 1, *p* = 0.042) (Fig. 4A). At pH 7.5 (weak nitrite toxicity), however, we found that the log r_S/G_s did not significantly change over the first three transfers (Mann–Kendall trend test; tau = 0.67, *p* = 0.31) (Fig. 4B). Thus, our data indicate that the generalist and specialist can indeed coexist, but only conclusively at pH 6.5 when the duration of nitrite availability is relatively long (Fig. 3).

We could simulate the observed co-culture dynamics using a two-species Monod-type model where the generalist consumes nitrate, produces conditionally toxic nitrite, and finally consumes nitrite, while the specialist only consumes nitrite. We parametrized the model using our available growth data (Supplementary Fig. S4) and assumed that the generalist and specialist have equivalent kinetics for nitrite consumption (with the exception that the specialist has no competitive interaction between the nitrate and nitrite reductases). We then assembled both cell types at the initial log r_S/G_s used in our experiments and simulated six serial transfers without adaptation of the model parameters.

The model accurately recreates the origin of the cross-feeding interaction where coexistence depends on nitrite toxicity (Fig. 4; dashed lines). The model suggests further factors that promote coexistence. In general, higher nitrate (and in turn higher nitrite) concentrations or conditions where nitrite is increasingly toxic (lower pH) accelerate the increase in the frequency of the specialist from rare (Supplementary Fig. S5). For example, at moderate nitrite toxicity, the specialist can increase in frequency from rare if sufficient nitrite is present (Fig. 3C; nitrite concentration correlates with intermediate durations of nitrite availability regardless of pH), but is likely outcompeted if the nitrite concentration is insufficiently high.

### Long-term dynamics of the nitrite (NO_2_^-^) cross-feeding interaction

We next evaluated the long-term dynamics of the nitrite cross-feeding interaction after its origin. Because we only experimentally observed coexistence at pH 6.5 (strong nitrite toxicity), all our further analyses focus on dynamics at this pH. We found that control cultures containing only the generalist displayed significantly reduced durations of nitrite availability after only three transfers (two-sample two-sided *t*-test, *p* = 0.003) (Supplementary Fig. S6). If the generalist altered its phenotype to reduce nitrite toxicity, then the emergence of such a phenotype may have important implications for the long-term persistence of the cross-feeding interaction (Fig. 1). We therefore serially transferred the co-cultures to a fresh medium for a total of 12 transfers. Our goal was to continue serial transfers such that genetic and phenotypic changes were more likely to emerge and accumulate.

We found that the long-term dynamics of the nitrite cross-feeding interaction depends on the initial composition of the co-culture (Fig. 4A). When the generalist was initially rare (measured initial log r_S/G_s of 3.53, 1.93, and 0.26), the composition persisted without any further statistically observable changes to the r_S/G_ between the third and twelfth transfers (Mann–Kendall trend tests; tau = –0.55 to 0.17, *p* > 0.6), and thus followed model predictions (Fig. 4A). However, when the specialist was initially rare (measured initial log r_S/G_s of –3.19, –2.65, and –0.88), the relative abundances of the specialist continuously decreased between the third and twelfth transfers (Mann–Kendall trend tests; tau = –0.61 to –0.89, *p* < 0.03), displaying a unimodal dynamic that deviated from model predictions (Fig. 4A). For example, when the measured initial log r_S/G_ was −3.19, the frequency of the specialist increased from 6.4 × 10^–4^ to 0.41 (640-fold) over the first three transfers, but then decreased to 8 × 10^–2^ (50-fold) over the remaining nine transfers (Fig. 4A and Supplementary Fig. S7). Overall, we observed a significant positive relationship between the initial and final log r_S/G_ after 12 transfers (two-sided Pearson correlation test; *r* = 0.87, *p* = 0.025, *n* = 6).

### Initial r_S/G_ controls long-term co-culture dynamics and the duration of nitrite availability

Why would the initial co-culture composition determine the long-term dynamics of the nitrite cross-feeding interaction? We hypothesized that the initial composition determines the initial environment, which in turn determines the initial selection pressures acting on the generalist. For example, if the initial frequency of the specialist were large, then there could be a sufficient specialist to rapidly consume all the nitrite released by the generalist. Nitrite would not be available to the generalist, eliminating any benefits to a generalist with improved nitrite consumption and promoting the persistence of the cross-feeding interaction. Indeed, this is what we experimentally observed (Fig. 4A). In contrast, if the initial frequency of the specialist were small, then there might not be a sufficient specialist to rapidly consume all the nitrite released by the generalist. Nitrite would then be available to the generalist, providing benefits to a generalist with improved nitrite consumption and potentially disrupting the cross-feeding interaction. Again, this is what we experimentally observed (Fig. 4A).

**Fig. 5:**
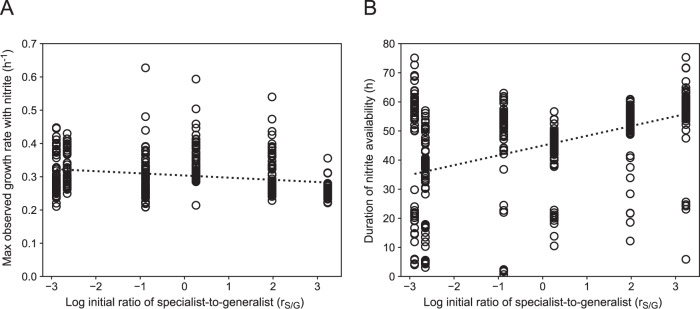
Growth properties of individual isolated generalists after 12 serial batch transfers of co-cultures at pH 6.5 (strong nitrite toxicity). We mixed the generalist and specialist together at different initial r_S/G_s and grew them at pH 6.5 under anoxic conditions with nitrate as the growth-limiting substrate. After 12 serial transfers, we isolated individual generalists and measured (**A**) the maximum growth rate with nitrite and (**B**) the duration of nitrite availability for each isolate. Data points are measurements for individual isolates. The dashed lines are linear regression models fit to the means for each initial r_S/G_.

If the above hypothesis were valid, then it leads to a clear expectation. As the initial r_S/G_ increases, the generalist is less likely to accumulate phenotypes with improved nitrite consumption traits. To test this, we isolated 70 individual generalists from six co-cultures initiated at different r_S/G_s (420 total isolates) after 12 serial transfers and measured the maximum observed growth rate with nitrite and the duration of nitrite availability. We did not observe a relationship between the initial log r_S/G_ and the maximum observed growth rate with nitrite (two-sided Pearson correlation test; *r* = −0.51, *p* = 0.30, *n* = 6) (Fig. 5A), but we did observe a significant positive relationship with the duration of nitrite availability (two-sided Pearson correlation test; *r* = 0.90, *p* = 0.014, *n* = 6) (Fig. 5B). Thus, generalists serially transferred in co-cultures with larger initial r_S/G_s are less likely to acquire significantly shorter durations of nitrite availability (Fig. 5B).

### Initial r_S/G_ controls the phenotypic diversification of the generalist

We finally tested whether the initial r_S/G_ determines the phenotypic changes in nitrite production and consumption acquired by the 420 individual generalist isolates. To test this, we delineated the individual generalist isolates based on their growth properties with nitrate and nitrite, including maximum observed cell densities, maximum observed growth rates, and the duration of nitrite availability. We provide the complete methods for delineating different individual-level phenotypes in the Supplementary Information.

Overall, we found that the generalist isolates displayed five distinct phenotypes (designated as phenotypes A–E) (Supplementary Fig. S8). Phenotype A immediately switched between nitrate and nitrite consumption (Supplementary Fig. S8A). Phenotype B had a short time delay between nitrate and nitrite consumption and its cell density remained constant during that time (Supplementary Fig. S8B). Phenotype C (ancestral phenotype) had a long time delay between nitrate and nitrite consumption and its cell density remained constant during that time (Supplementary Fig. S8C). Phenotype D had a short time delay between nitrate and nitrite consumption and its cell density declined during that time (Supplementary Fig. S8D). Phenotype E had a long time delay between nitrate and nitrite consumption and its cell density declined during that time (Supplementary Fig. S8E). We further used genome re-sequencing of five or six randomly chosen representatives of each phenotype (A–E) to test whether these phenotypes correlate with genetic changes. We found that all isolates displaying phenotypes A, D, and E had genetic changes while all isolates displaying phenotypes B and C did not (Supplementary Table S2). Thus, our data suggest that phenotypic diversification of the generalist likely occurred via both genetic and non-genetic changes.

**Fig. 6:**
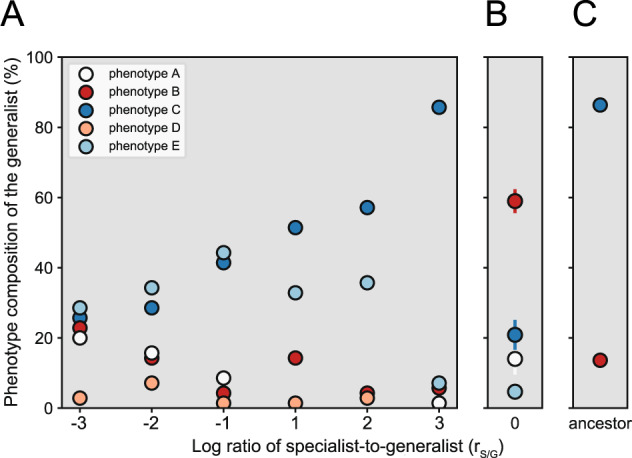
Phenotype composition of the generalist population after 12 serial batch transfers at pH 6.5 (strong nitrite toxicity). After 12 serial transfers, we isolated individual generalists from one experimental series and assigned each isolate to a phenotype group based on its growth pattern (see the Supplementary Information). **A** Generalist isolates from co-cultures with initial log r_S/G_s > 0 were dominated by phenotype C (dominant ancestral phenotype with a long time delay between nitrate and nitrite consumption), while generalist isolates from co-cultures with initial r_S/G_s < 0 had significant proportions of phenotypes A, B, and D (short or no time delay between nitrate and nitrite consumption). Data points are individual measurements. **B** The generalist transferred alone was dominated by phenotype B (short time delay between nitrate and nitrite consumption). Data points are means and error bars are one standard deviation of four biological replicates. **C** Some ancestral generalist isolates displayed phenotype B while the majority displayed phenotype C.

We observed relationships between the initial r_S/G_s and the frequencies of specific phenotypes acquired by the generalist (Fig. 6). Phenotype A decreased in frequency as the initial r_S/G_ increased (two-sided Pearson correlation test; *r* = −0.94, *p* = 6.2 × 10^–3^, *n* = 6) (Fig. 6). This phenotype has no observable time delay between nitrate and nitrite consumption (Supplementary Fig. S8A). Thus, as the specialist became dominant, generalists that immediately switched from nitrate to nitrite consumption became scarce. Conversely, phenotype C increased in frequency as the initial r_S/G_ increased (two-sided Pearson correlation test; *r* = 0.95, *p* = 4.2 × 10^–3^, *n* = 6) (Fig. 6). This phenotype has the longest time delay between nitrate and nitrite consumption (Supplementary Fig. S8C). Thus, as the specialist became dominant, generalists with a long time delay between nitrate and nitrite consumption became dominant. Taken together, our data indicate that the initial r_S/G_ controls the phenotypic diversification and trajectory of the generalist.

### Initial r_S/G_ determines the fitness gains of evolved generalist phenotypes

**Fig. 7:**
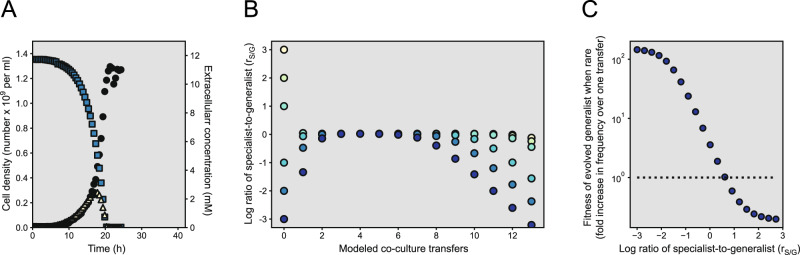
Evolution of the generalist disrupts the cross-feeding interaction. **A** We grew phenotype A of the generalist alone in a bioreactor at pH 6.5 (strong nitrite toxicity) under anoxic conditions with nitrate as the growth-limiting substrate. Blue squares are measured extracellular nitrate concentrations, yellow triangles are measured extracellular nitrite concentrations, and black circles are measured cell densities. We measured extracellular nitrate and nitrite concentrations with IC and cell densities with FC. **B** In a three-species model, we introduced phenotype A of the generalist at a low initial frequency (1 × 10^–8^) and varied the initial r_S/G_. The model assumes that after each growth cycle, a small proportion of the ancestral generalist (1 × 10^–8^) mutates into phenotype A. Symbols show computed results at the end of each simulated transfer. **C** Using the three-species model, we introduced phenotype A of the generalist at a low initial frequency (1 × 10^–8^) and varied the initial r_S/G_. Symbols are the fold-increase in the frequency of phenotype A over one transfer. The dotted line separates the results depending on whether phenotype A has positive fitness (above the line) or negative fitness (below the line).

Can the presence of generalist phenotypes with altered nitrite consumption traits explain the displacement of the specialist after cross-feeding is fully established? To test this, we grew generalist phenotype A alone in bioreactors with anoxic ACS medium amended with 12 mM nitrate and measured the extracellular concentrations of nitrate and nitrite over time (Fig. 7A). In contrast to the ancestral generalist (Fig. 3A, B), phenotype A consumed nitrate and nitrite simultaneously and recovered the cell yield per unit substrate consumed (Fig. 7A and Supplementary Fig. S4E, F). We could describe phenotype A with a model where competitive inhibition between nitrate and nitrite is relaxed (Supplementary Fig. S4E, F). We then combined it with models describing the ancestral generalist (phenotype C) and the specialist and simulated serial transfers (Fig. 7B). This time, we allowed a small proportion (1 × 10^–8^) of the ancestral generalist to transform (mutate) into phenotype A prior to each transfer. Enabling the evolution of phenotype A resulted in dynamics similar to those that we observed experimentally (Fig. 4A and 7B). Thus, accounting for evolution resolved discrepancies between experiments and model simulations at small initial r_S/G_s (Fig. 4A and 7B). We finally asked how the frequency of the specialist affects the fitness of phenotype A. As long as the frequency was larger than that of the generalist, the fitness of phenotype A relative to the ancestral generalist was below 1 (Fig. 7C), conditions that we only observed during the first transfer (Fig. 4A). This further explains the reduction or absence of phenotype A after 12 transfers in co-cultures that were initiated at large r_S/G_s (Fig. 6).

## Discussion

We demonstrated that initial co-culture composition determines the dynamics, phenotypic diversification, and long-term persistence of a cross-feeding interaction. In our case, large initial r_S/G_s result in convergence to a consistent co-culture composition (at least over the experimental timecourse) while small initial r_S/G_s result in increasing dominance of the generalist (Fig. 4). Why did we observe these different dynamics? Our data and mathematical models support the hypothesis that initial co-culture composition sets the early environmental selection pressures, which in turn directs the future phenotypic trajectory of the cross-feeding interaction (Figs. 5 and 7). High initial r_S/G_s have high specialist frequencies. Nitrite will therefore be largely unavailable to the generalist after nitrate is completely consumed. This reduces selection strength for generalist phenotypes with improved nitrite consumption and consequently enables long-term persistence of the cross-feeding interaction (i.e., mutations that improve nitrite reduction are less likely to be among the most beneficial mutations, and thus less likely to overcome clonal interference). In contrast, low initial r_S/G_s have high generalist frequencies. Nitrite will therefore remain available to the generalist after nitrate is completely consumed. This increases selection strength for generalist phenotypes with improved nitrite reduction (i.e., mutations that improve nitrite reduction are more likely to be among the most beneficial mutations, and thus more likely to overcome clonal interference). Ultimately, this could cause the generalist to displace the specialist, disrupt the cross-feeding interaction, and deteriorate biodiversity (Fig. 7A, B). Thus, the early environment not only determines the initial selection pressures and phenotypic trajectories, but also has cascading effects on the subsequent environment, the long-term persistence of the interaction, and the maintenance of biodiversity.

An alternative explanation for our main outcome is that the different dynamics were caused by different initial population sizes rather than by different initial community compositions. In our experiments, we varied the initial r_S/G_ while keeping the initial total cell density constant across co-cultures (Fig. 4). One consequence is that as the r_S/G_ decreases, the size of the generalist population increases, which in turn increases the total load of potential mutations within the generalist population. This could affect the amount of standing genetic variation for selection to act upon, and thus modify the pace and trajectory of evolution.

We have three arguments for why differences in population sizes alone, however, are unlikely to explain our main results. First, all of the co-cultures rapidly converged to the same r_S/G_ within the first few transfers (Fig. 4). There were therefore no substantial differences in population sizes for the majority of the experiment. If population sizes were the primary force driving phenotypic trajectories, we would expect the distribution of generalist phenotypes to be similar across treatments at the end of the experiment, which we did not observe (Fig. 6). Second, the number of generalist cells grown during each batch transfer was similar between co-cultures and two orders of magnitude higher than in the inoculum. We also inoculated all the co-cultures with the same generalist population that, prior to inoculation, was grown aerobically. While we cannot exclude some genetic variation in the generalist inoculum, we can exclude the preferential selection of mutants with improved nitrite consumption prior to inoculation. In addition, if phenotype A (immediate switching between nitrate and nitrite reduction) was indeed introduced with the inoculum, then we would expect that the generalist isolates displaying phenotype A would all have the same genetic background regardless of the initial r_S/G_, which was not the case. Each of the five sequenced generalist isolates displaying phenotype A acquired different genetic changes and emerged in different co-cultures, and therefore likely arose independently after initiation of the experiment. Third, we performed additional simulations where we varied the initial r_S/G_ while fixing the initial population size of the generalist or specialist (Supplementary Fig. S9). We observed quantitative differences with our previous modeling where we did not fix the initial population sizes (Fig. 7B). However, our qualitative conclusion remains valid, as the initial r_S/G_ nevertheless affects the dynamics and persistence of the cross-feeding interaction (Supplementary Fig. S9). Thus, our data indicate that initial community composition itself can be an important determinant of the dynamics and long-term persistence of cross-feeding interactions.

An open question in microbial community assembly is why community composition is so temporally dynamic, even on relatively simple substrates such as polysaccharide particles (e.g., marine snow [36]). Our results offer a plausible mechanism to explain why high taxonomic turnover is observed in these systems. As community succession proceeds, the abundance ratios of metabolically interacting cell-types changes [37], which might lead to the rapid extinction of specialist cell types depending on such abundance ratios. By modulating niche availability, these ratios can play a crucial role in allowing the recruitment of new specialist types to the community due to the extinction of former specialist cell types, leading to high taxonomic turnover while retaining functional stability. Whether the high competition for secondary metabolites in these systems diminishes the duration of specialized niches, and thus the phenotypic diversification of generalist types, remains to be tested.

We believe our results may also be relevant for understanding the role of priority effects on the maintenance of biodiversity across communities [38, 39]. Consider that the dispersion of cells into new environments is, at least to some extent, a stochastic process. If the generalist and specialist are randomly dispersed across a surface, then there will be variation in the local initial r_S/G_s across space. This, in turn, could constrain the local dynamics and phenotypic trajectory of the cross-feeding interaction. The outcome would be a diversity of local communities, where each evolves in response to the local resource niche determined by the local initial r_S/G_. Systems such as the human gut, where it is still unclear what causes high variation in community composition among individual hosts [38], could be driven by such variations across space.

Our results may also have important implications for microbial biotechnology. There is increasing interest in applying synthetic microbial consortia to achieve engineering objectives [7, 40–44]. Important advantages of synthetic microbial consortia are that (a) they can be designed in a rational manner based on the traits of available strains and the desired engineering objectives, and (b) they have reduced complexity relative to natural microbial communities that enables improved engineering control [7, 40–44]. When designing synthetic microbial consortia, one decision point is the initial frequencies of the component strains. Here, we demonstrate that the selection of initial frequencies can have profound effects on the long-term dynamics and trajectory of a microbial community. Thus, information about the traits of the component strains may be insufficient alone to select the most appropriate initial frequencies, and further information about how the initial frequencies affect the environment and the selection pressures acting on the component strains may also be needed.

## Supplementary information

Supplementary Material

## Data Availability

All experimental data and codes used in this study are publicly available in the Eawag Research Data Institutional Collection (ERIC) repository (10.25678/0006RZ). All sequence data are publicly available in the NCBI Sequence Read Archive (https://www.ncbi.nlm.nih.gov/sra) under BioSample accessions SAMN20157770–SAMN20157812.
