# Supplementary material for: Initial community composition determines the long-term dynamics of a microbial cross-feeding interaction by modulating niche availability
Source: ISME Commun. 2022 Aug 24;2:77. doi: 10.1038/s43705-022-00160-1 (PMC9723679; doi:10.1038/s43705-022-00160-1)
Supplement: Supplementary file 1 — Supplementary Material [file 43705_2022_160_MOESM1_ESM.pdf]

Supplementary Material

Initial community composition determines the long-term co-existence of microbial cross-feeding cell-types by modulating niche availability

Jan Dolinšek[1,2],Josep Ramoneda[1,3], David R. Johnson[1,*]

[1]Department of Environmental Microbiology, Swiss Federal Institute of Aquatic Science and Technology (Eawag), 8600 Dübendorf, Switzerland; [2]Department of Environmental Systems Science, Swiss Federal Institute of Technology (ETH), 8092 Zürich, Switzerland; [3]Cooperative Institute for Research in Environmental Sciences, University of Colorado, Boulder, CO, USA.

* Corresponding author (david.johnson@eawag.ch)

This file includes:

Supplementary Figures S1-S9

Supplementary Tables S1-S2

Supplementary Materials and Methods

Supplementary Results

Supplementary References

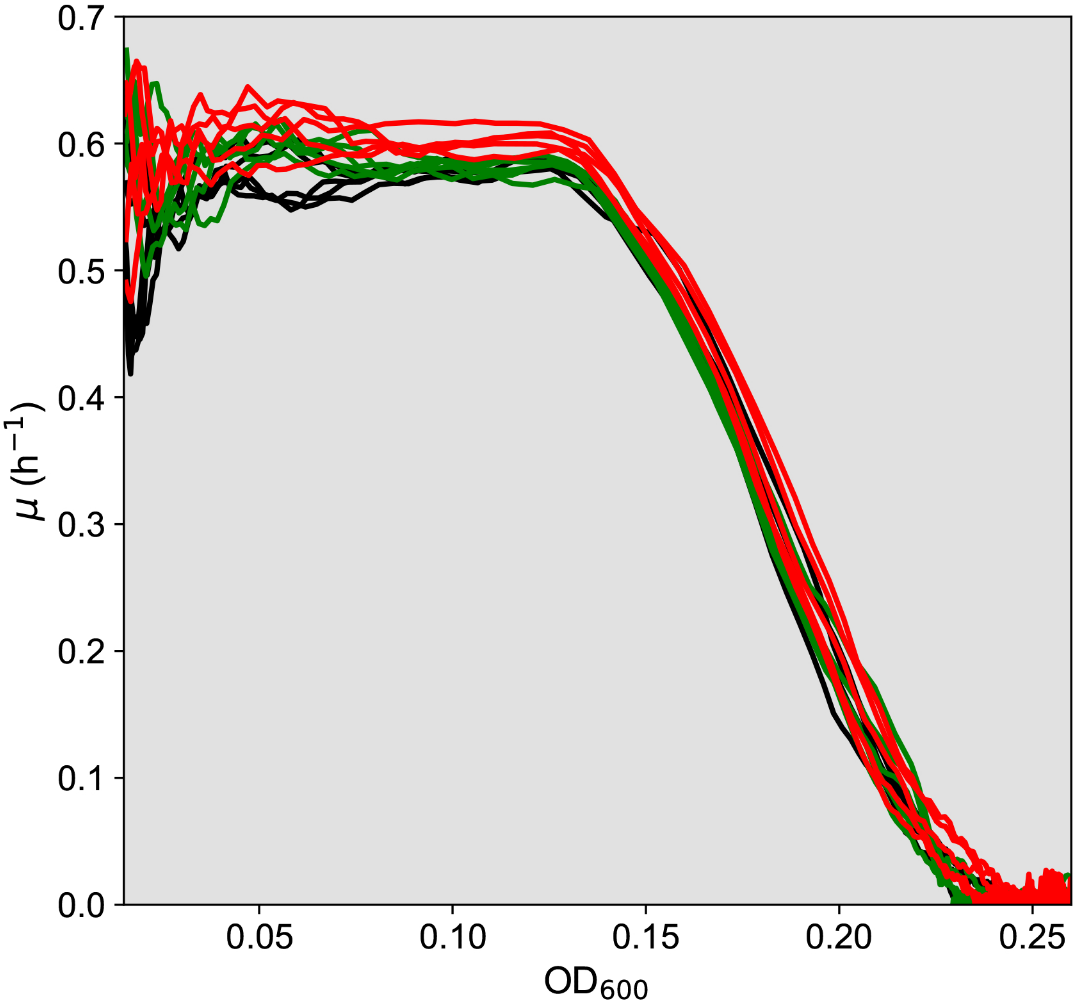

Supplementary Fig. S1: Measured growth rates (*μ*) of the individual ancestral strains of *Pseudomonas stutzeri* in oxic ACS medium as a funciton of cell density (OD_{600}). We grew the generalist (black lines), specialist carrying the *egfp* gene (green lines), and specialist carrying the *echerry* gene (red lines) alone in batch culture for 24 hours in a 96-well microtitre plate. Each line is for an independent replicate.

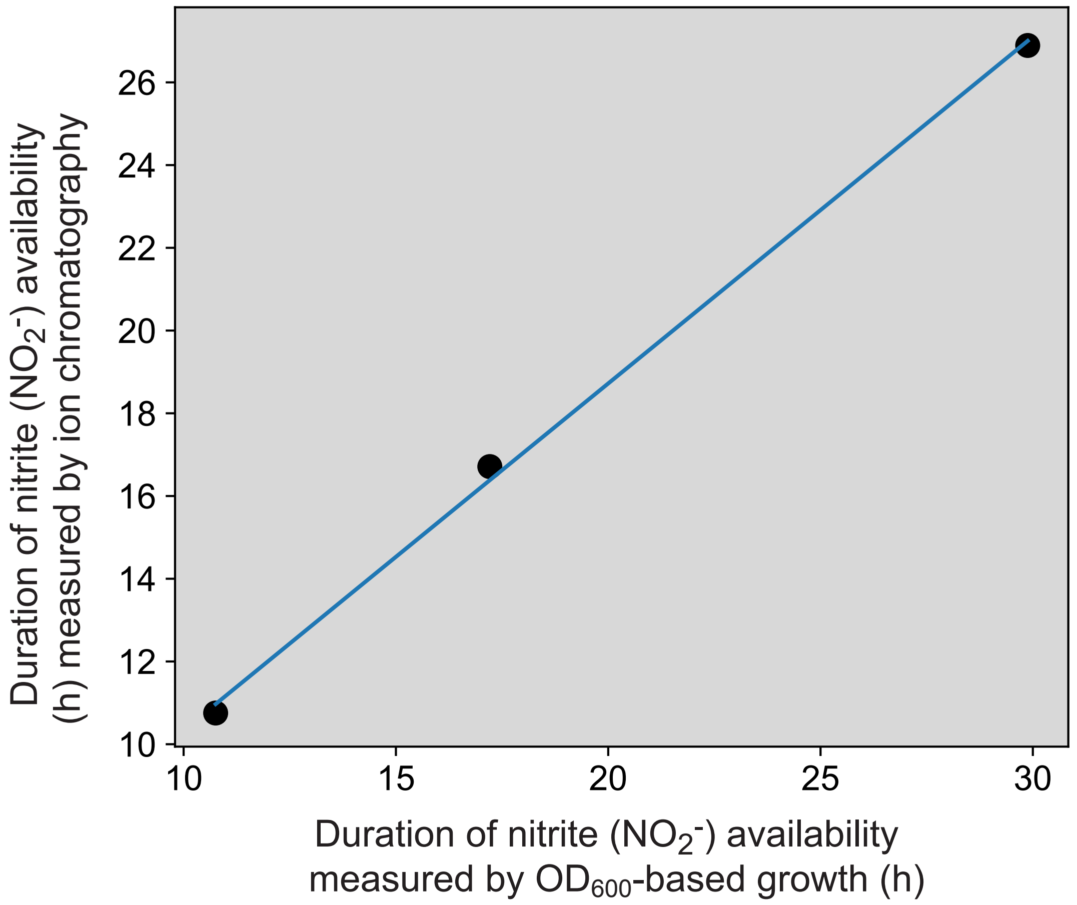

Supplementary Fig. S2: Comparison of the growth-based proxy measures and direct ion chromatography-based measures of the duration of nitrite (NO_2^-) availability. We grew the generalist alone in anoxic bioreactors with varying amounts of nitrate (NO_3^-) and at different pH conditions. Left data point, 12 mM nitrate at pH 7.5; middle data point, 5 mM nitrate at pH 6.5; right data point, 12 mM nitrate at pH 6.5. The solid line is a linear regression model fit to the data.

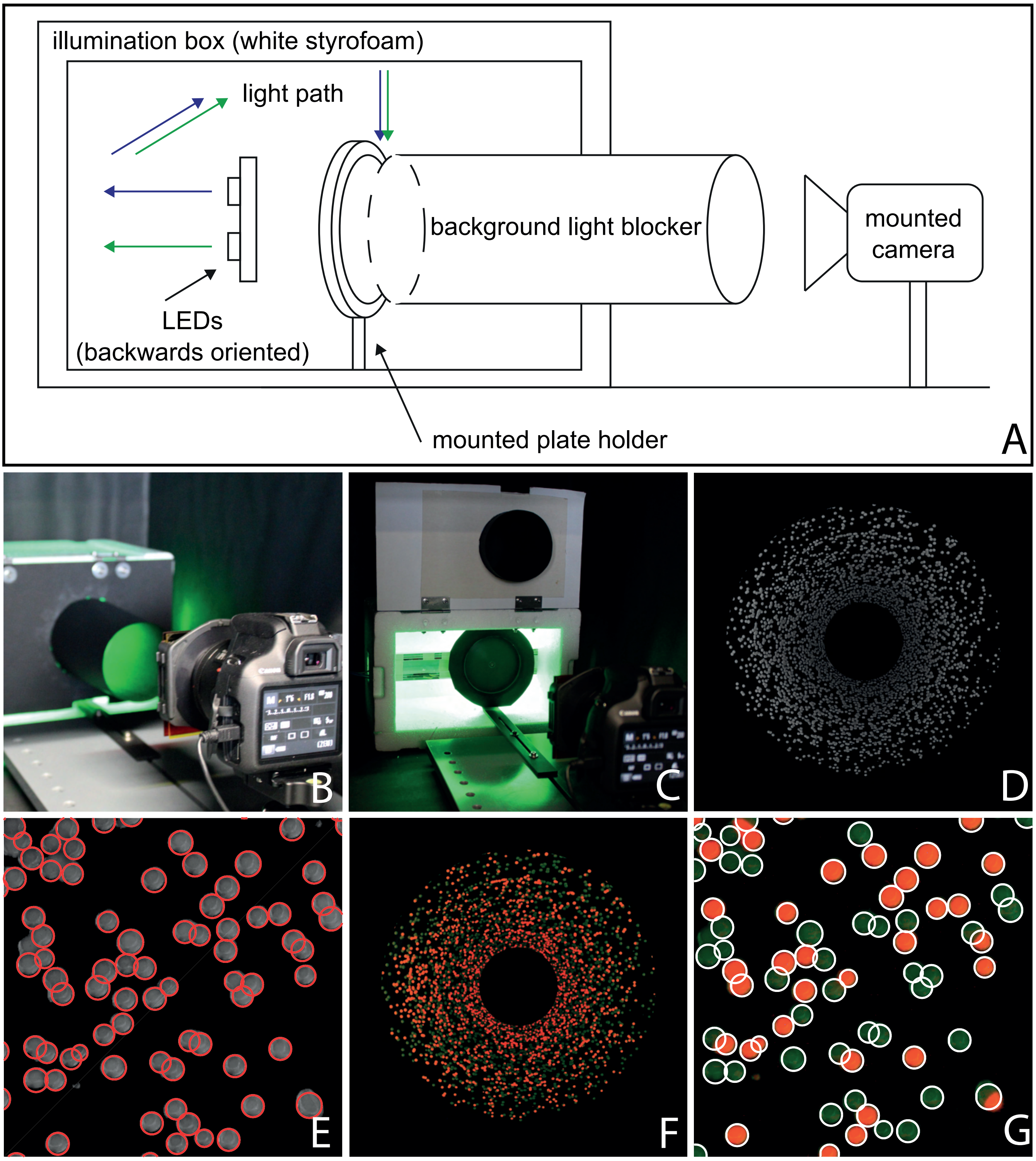

Supplementary Fig. S3: Fluorescence photography setup and analysis. A Schematic illustration and **B**, **C** photographs of our setup used to capture fluorescent images of colonies on agar plates. **D** We first converted reflected light photographs of all colonies into grayscale and removed non-relevant image areas (*e.g.*, the overgrown central area of the agar plate or the area outside petri dish) and background pixels. **E** We then identified circular objects within a predefined parameter range (radius range, sensitivity factor) from the reflected light image. We finally combined **F** the fluorescent image with **G** the identified circular objects to quantify frequencies of colonies expressing *egfp* or *echerry*.

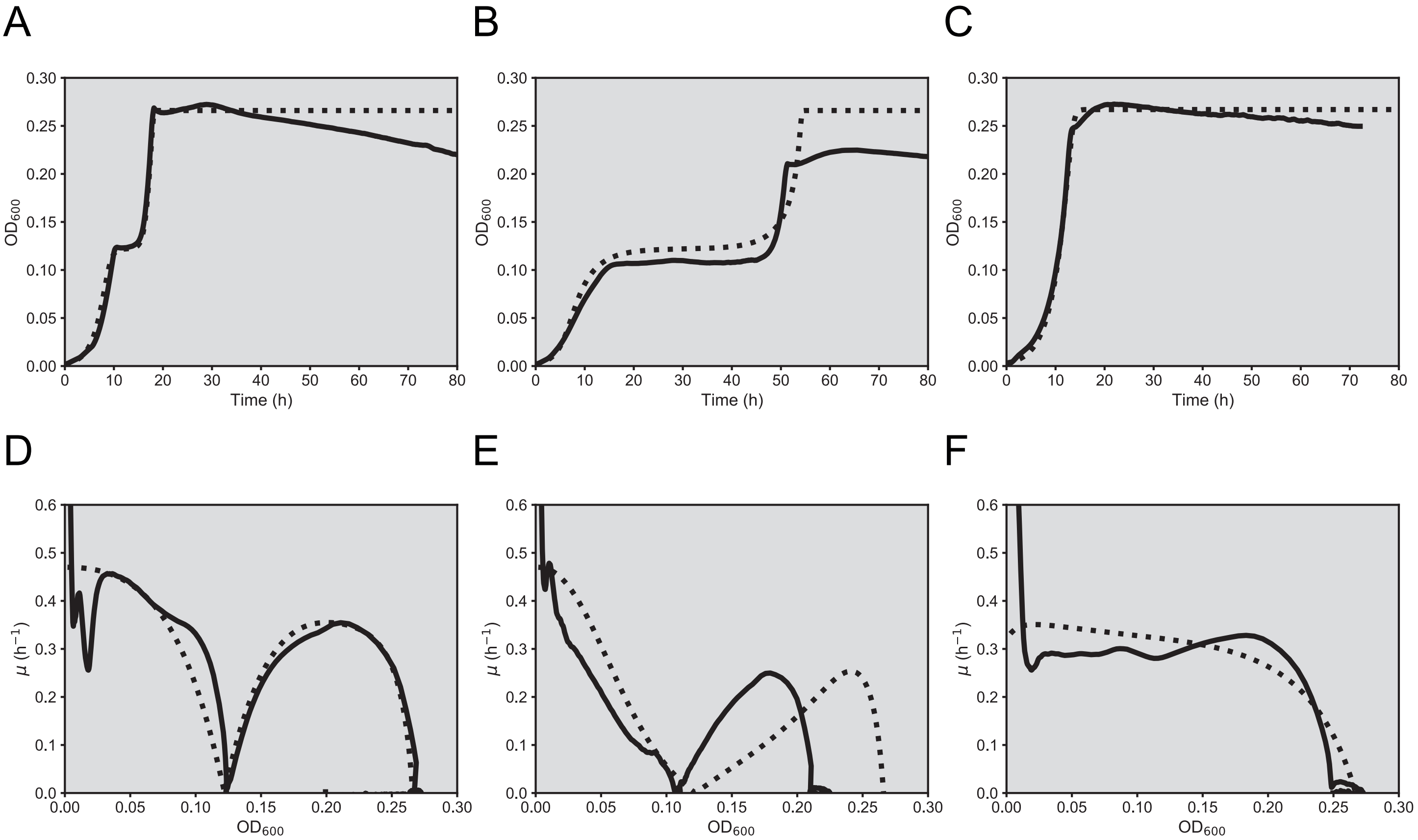

Supplementary Fig. S4: Generalist growth model calibration. We grew the ancestral generalist and phenotype A of the generalist alone in batch culture in microtiter plates. We used OD_{600} data to calibrate the growth yield, maximum growth rate, and intermediate toxicity parameters of our dynamical model. We first calibrated the model with data from the ancestral generalist grown at pH 7.5 (weak nitrite [NO_2^-] toxicity). Our objective was to describe growth under different conditions (nitrite toxicity) or of different phenotypes (phenotype A) with as few parameter changes as possible, rather than to make the best possible model fit to the measured data for each scenario. Solid lines are the measured OD_{600} data and dotted lines are model predictions. **A**, **B** Ancestral generalist grown at pH 7.5 (weak nitrite toxicity). **C**, **D** Ancestral generalist grown at pH 6.5 (strong nitrite toxicity). **E**, **F** Phenotype A of the generalist grown at pH 6.5 (strong nitrite toxicity).

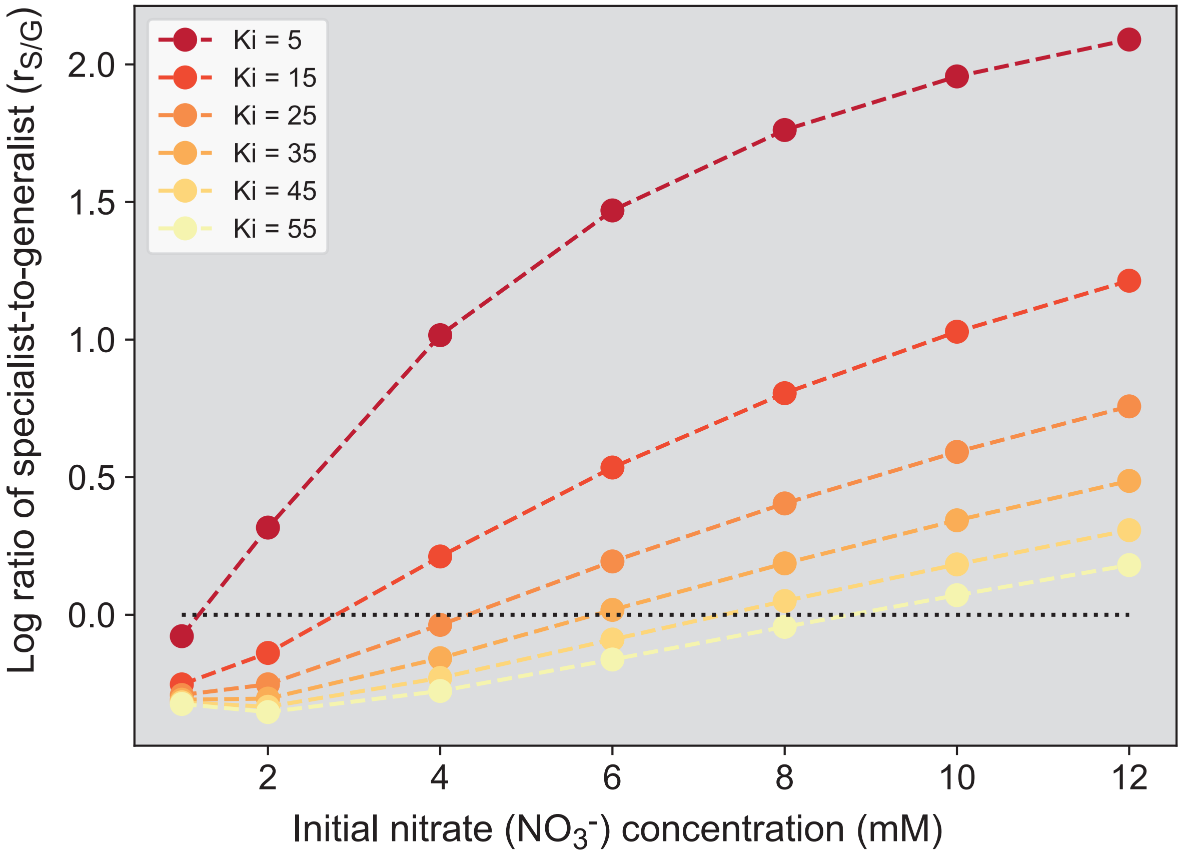

Supplementary Fig. S5: Combination of intermediate concentration and intermediate toxicity determines the ability of the specialist to increase in frequency when rare. The mathematical model shows that for any set of tested intermediate toxicity parameters, the metabolic intermediate concentration is a major determinant controlling wether the specialist can increase in freqeuncy when rare and establish a cross-feeding interaction. Circles show the increase/decrease in $\log_{10}$ transformed $r_{S/G}$. The dotted line separates scenarios where the specialist increases in frquency or is displaced by the generalist.

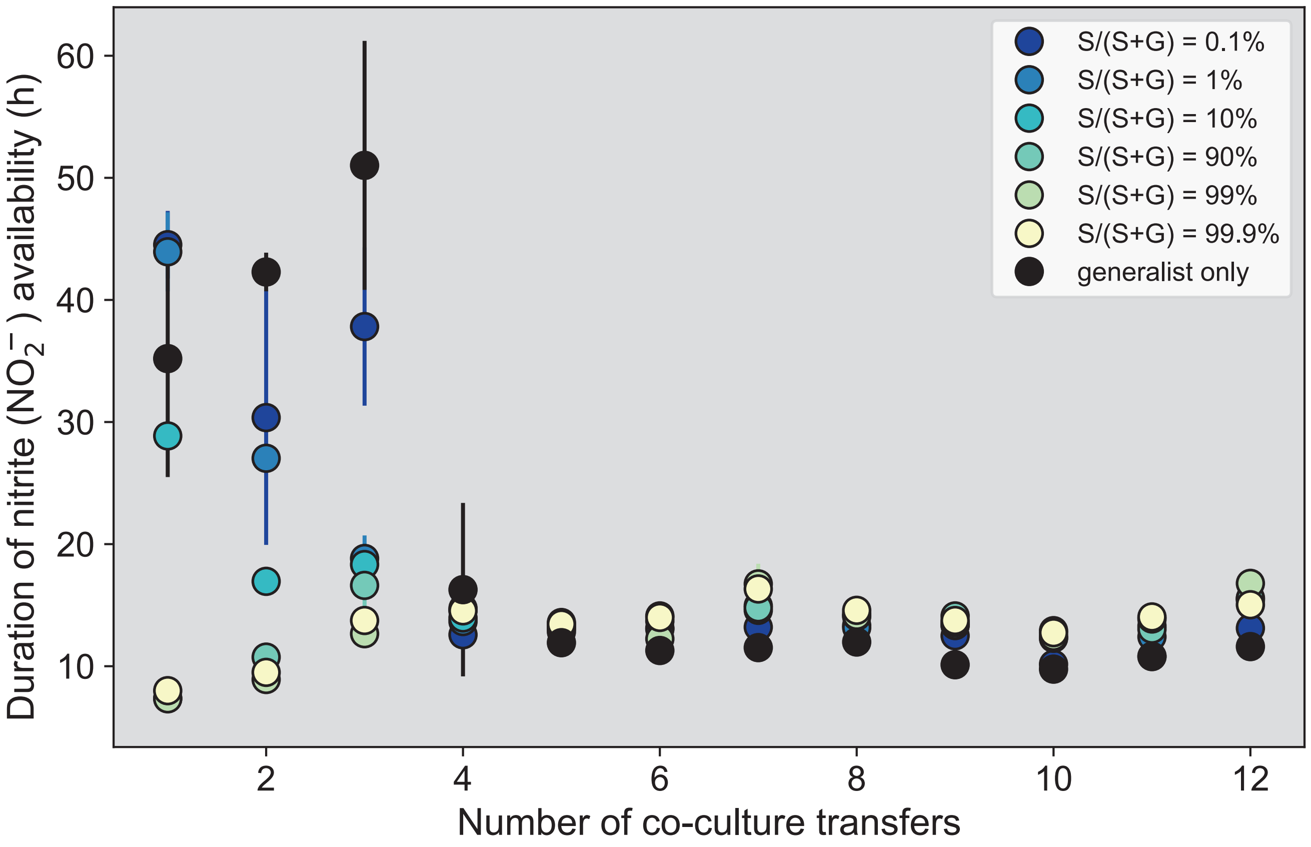

Supplementary Fig. S6: Dynamics of the duration of nitrite (NO_2^-) availability during serial transfer in anoxic ACS medium. We mixed the generalist and specialist togeter at the indicated initial frequencies and serially transferred them with nitrate (NO_3^-) in batch cultures at pH 6.5 (strong nitrite toxicity). Colored symbols are means and colored error bars are one standard error of two biological replicates. Black symbols are means and black error bars are one standard deviation of eight biological replicates (generalist-only controls).

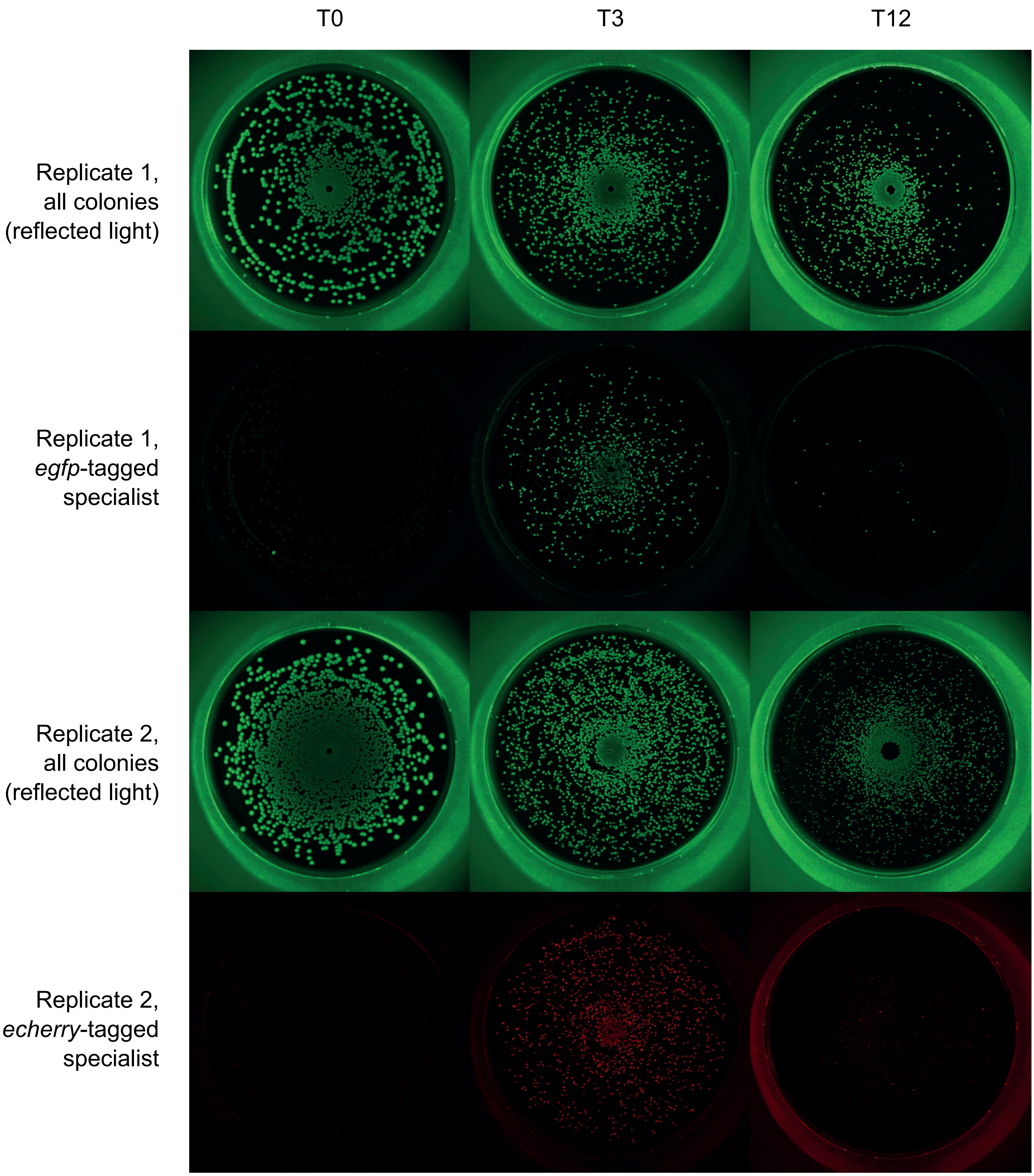

Supplementary Fig. S7: Specialists increase and decrease over time when initially rare at pH 6.5 (strong nitrite [NO_2^-] toxicity). We initiated co-cultures with a specialist frequency of approximately 0.1% and acquired fluorescent photographs of spiral streaks from the co-cultures. The left column images are of plates streaked from the initial specialist/generalist mixture (T0). The center column images are of plates streaked from co-cultures after three transfers (T3). The right column images are of plates streaked from co-cultures after twelve transfers (T12). The first and third rows are reflected light images showing both specialist and generalist colonies while the second and fourth rows are the respective fluorescent light images (image of the same agar plate) where the specialist colonies are fluorescent and the generalist colonies are not. The first and second rows show one replicate co-culture where the specialist is tagged with *egfp*, while the third and fourth rows show a second replicate co-culture where the specialist is tagged with *echerry*.

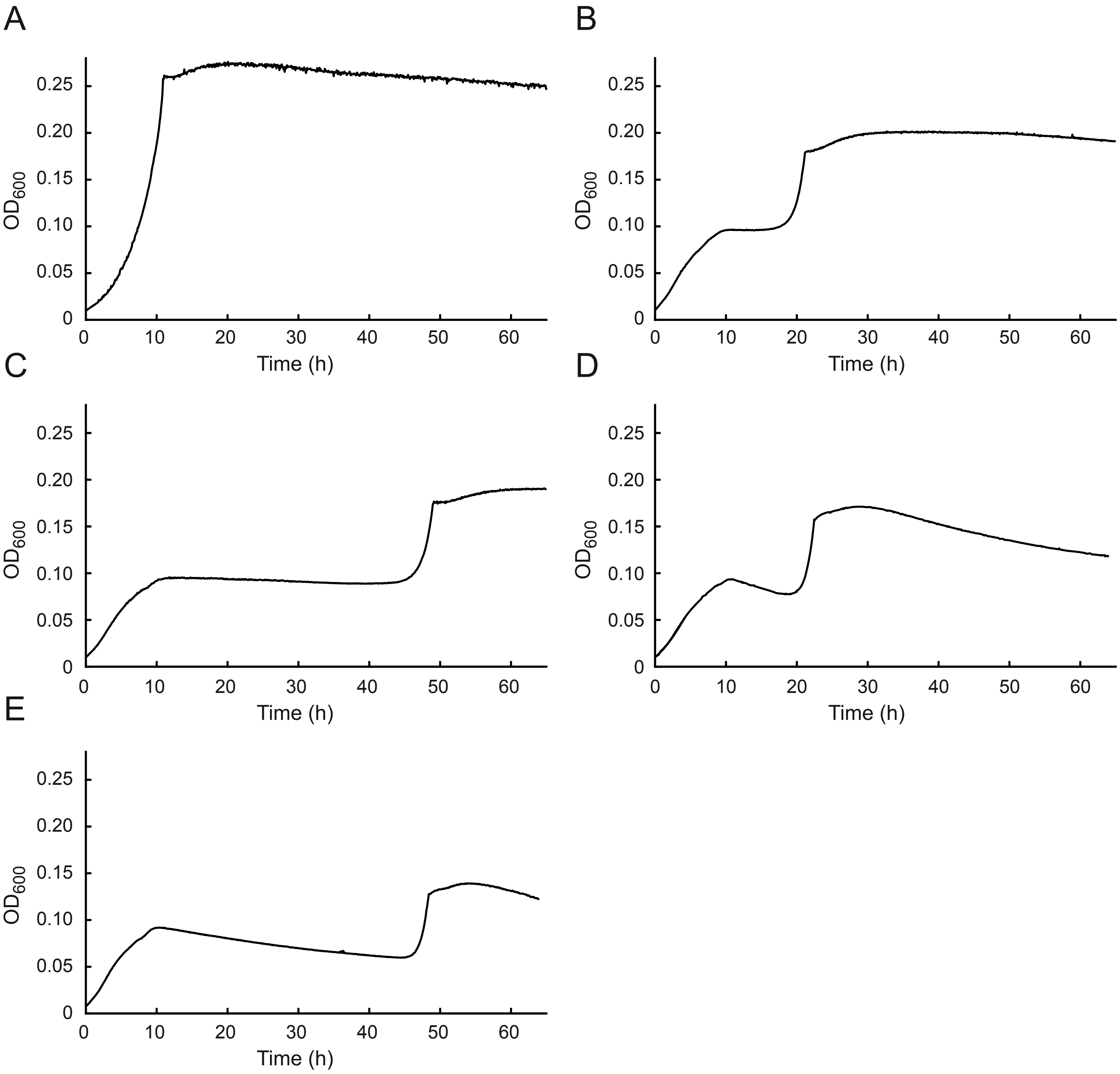

Supplementary Fig. S8: Growth of individual isolated generalists after twelve serial batch transfers of co-cultures at pH 6.5 (strong nitrite [NO_2^-] toxicity). We grew individual generalist isolates alone in batch culture, and these generalist isolates displayed five distinct phenotypes. **A** Phenotype A immediately switches between nitrate (NO_3^-) and nitrite reduction, **B** phenotype B has a short time delay between nitrate and nitrite reduction, **C** phenotype C has a long time delay between nitrate and nitrite reduction, **D** phenotype D has a short time delay between nitrate and nitrite reduction and its cell density declines during that time, and **E** phenotype E has a long time delay between nitrate and nitrite reduction and its cell density declines during that time. A detailed description of how we delineated the phenotypes is provided in the Supplementary Text.

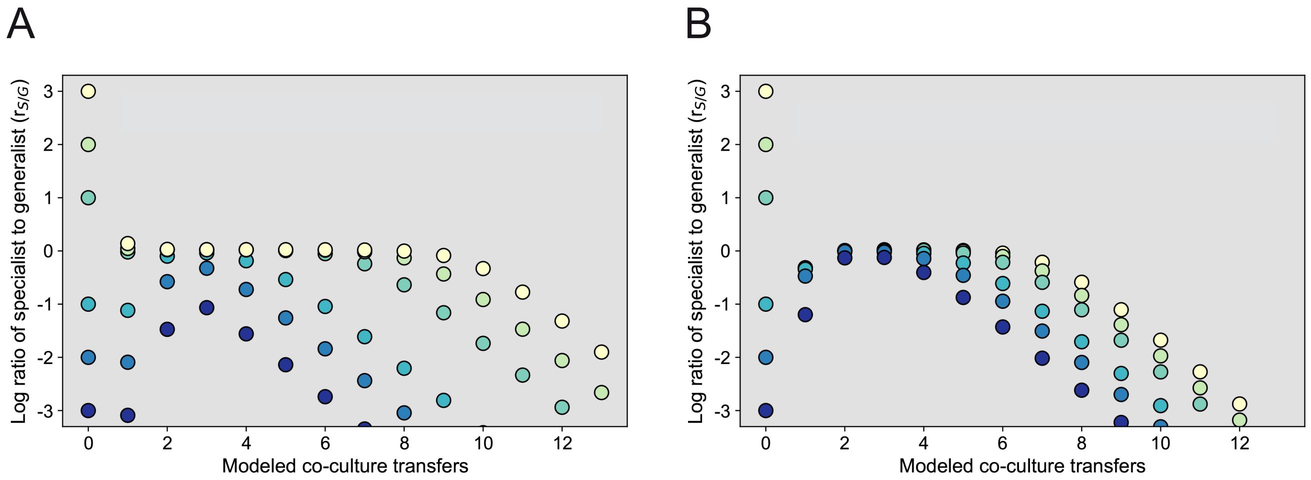

Supplementary Fig. S9: Model simulations for co-cultures of the generalist and specialist initiated at different $r_{S/G}$s. We varied the initial $r_{S/G}$ while holding the initial population size of **A** the generalist or **B** the specialist constant. The simulations are otherwise identical to those presented in Fig. 7.

Supplementary Table S1. Isogenic mutant strains of *Pseudomonas stutzeri* A1501 used in this study.

Strain	Relevant characteristics
**Generalist strain**	
A1601	*P. stutzeri* A1501 with Δ*comA* and mini-Tn7T-LAC-Gm; Gm^R, defective in competence
**Specialist strains**	
A1602gfp	*P. stutzeri* A1501 with Δ*comA*, Δ*narG* and mini-Tn7T-LAC-Gm-*egfp*; Gm^R, $egfp^+$, defective in growth with nitrate as an electron acceptor, defective in competence
A1602ech	*P. stutzeri* A1501 with Δ*comA*, Δ*narG* and mini-Tn7T-LAC-Gm-*echerry*; Gm^R, $echerry^+$, defective in growth with nitrate as an electron acceptor, defective in competence

Supplementary Table S2. Genetic changes acquired by the generalist after 12 serial co-culture transfers at pH 6.5 (strong nitrite toxicity).

Clone	Selection environment	Phenotype	Genome position	Genetic change	Annotation	Gene	Description
1CT12_1B	Generalist only	A	997,181	Δ1 bp	coding (446/1488 nt)	PST_RS04580	MFS transporter
1CT12_1B	Generalist only	A	1,194,876	G→A	G11S (GGC→AGC)	*ftsQ*	Cell division protein FtsQ
1CT12_1B	Generalist only	A	2,746,178	G→A	intergenic (-536/-52)	PST_RS12620	Hypothetical protein/FAD-dependent oxidoreductase
9CT12_1A	1% Specialist	A	111,488	$(TGCTGG)_{2\to3}$	coding (151/702 nt)	PST_RS00490	Cytochrome B
9BT12_1A	0.1% Specialist	A	997,476	C→G	Y247* (TAC→TAG)	PST_RS04580	MFS transporter
9GT12_9F	99.9% Specialist	A	995,065	G→A	M140I (ATG→ATA)	PST_RS04570	NarK/NasA family nitrite transporter
9DT12_4F	10% Specialist	A	997,254	G→A	W173* (TGG→TGA)	PST_RS04580	MFS transporter
1CT12_1E	Generalist only	B	1,266,672	C→T	Q403Q (CAG→CAA)	PST_RS05890	Membrane protein
9DT12_6F	10% Specialist	C	246,464	T→C	K223R (AAG→AGG)	*cysT*	Sulfate ABC transporter permease subunit
9FT6_9D	99% Specialist, transfer 6	not determined	2,812,161	$(GCAGGCGGC)_{1\to2}$	coding (1214/1785 nt)	*fliF*	Flagellar M-ring protein
9FT12_3B	99% Specialist	D	2,812,161	$(GCAGGCGGC)_{1\to2}$	coding (1214/1785 nt)	*fliF*	Flagellar M-ring protein
9FT12_3B	99% Specialist	D	3,911,442	G→T	E77* (GAG→TAG)	PST_RS18040	Hypothetical protein
9BT12_4C	0.1% Specialist	D	2,797,032	G→A	R146C (CGC→TGC)	*flhA*	Flagellar biosynthesis protein
9ET12_2G	90% Specialist	D	1,487,407	new junction evidence	coding (326/741 nt)	*flgF*	Flagellar basal body rod protein
1CT12_2F	Generalist only	D	1,485,429	new junction evidence	coding (565/681 nt)	*flgD*	Flagellar basal body rod modification protein
1CT12_2F	Generalist only	D	1,584,384 - 1587032	new junction evidence	pseudogene (1068/1068 nt) pseudogene (22/1209 nt)	PST_RS07495 + PST_RS07510	Hypothetical protein
1CT12_2F	Generalist only	D	2,595,106	new junction evidence	coding (89/318 nt)	PST_RS21315	Hypothetical protein
9CT12_8E	1% Specialist	D	2,798,501	new junction evidence	coding (872/1137 nt)	*flhB*	Flagellar biosynthesis protein
9CT12_9B	1% Specialist	D	1,485,641	new junction evidence	coding (74/1317 nt)	*flgE*	Flagellar hook protein
9ET12_1B	90% Specialist	E	1,485,258	new junction evidence	coding (394/681 nt)	*flgD*	Flagellar basal body rod modification protein
1CT12_1G	Generalist only	E	1,484,940	new junction evidence	coding (76/681 nt)	*flgD*	Flagellar basal body rod modification protein
9CT12_7F	1% Specialist	E	1,486,074	new junction evidence	coding (507/1317 nt)	*flgE*	Flagellar hook protein
9BT12_5F	0.1% Specialist	E	713,273	new junction evidence	coding (509/771 nt)	PST_RS03215	NERD nuclease
9BT12_5F	0.1% Specialist	E	3,736,300	new junction evidence	coding (76/2874 nt)	PST_RS17210	ATP-dependent helicase
9BT12_5F	0.1% Specialist	E	2,772,673	new junction evidence	intergenic (-109/+278)	PST_RS12780	Fis family transcriptional regulator
9DT12_1G	10% Specialist	E	1,485,698	new junction evidence	coding (131/1317 nt)	*flgE*	Flagellar hook protein
9FT12_1A	99% Specialist	E	640,285	C→T	Q157* (CAG→TAG)	PST_RS02935	Hypothetical protein
9FT12_1A	99% Specialist	E	1,485,258	new junction evidence	coding (394/681 nt)	*flgD*	Flagellar basal body rod modification protein
9GT12_3G	99.9% Specialist	E	577,039	Δ1 bp	coding (962/1149 nt)	PST_RS02675	NnrS family protein
9GT12_3G	99.9% Specialist	E	2,015,873	new junction evidence	coding (26/387 nt)	*fliS*	Flagellar protein
9GT12_3G	99.9% Specialist	E	3,417,460	new junction evidence	coding (569/2619 nt)	PST_RS15900	Helix-turn-helix transcriptional regulator
9GT12_3G	99.9% Specialist	E	1,584,384 - 1587032	new junction evidence	pseudogene (1067/1068 nt) pseudogene (22/1209 nt)	PST_RS07495 + PST_RS07510	Hypothetical protein

Supplementary Materials and Methods

Bioreactor experiment. To characterize the growth and nitrogen oxide reduction properties of the generalist, we grew the generalist in 1 L continuously-stirred gas-tight culture flasks at 34°C (referred to as bioreactors). We first purged 445.5 mL of a completely defined synthetic citrate-asparagine medium (ACS medium) [1] amended with 5 mM or 12 mM sodium nitrate ($NaNO_3$), 10 mg/L gentamycin, and 1 mM sodium bromide (NaBr) with an anoxic nitrogen (N_2):hydrogen (H_2) (97:3) gas mixture while stirring for 45 minutes. This depleted dissolved oxygen in the medium to below the detection limit (0.3 µM) of our oxygen microsensor (Unisense, Aarhus, Denmark). We next inoculated the medium with 4.5 mL of an overnight culture of the generalist grown in oxic ACS medium. After inoculating the bioreactor, we continuously sampled small aliquots from the bioreactor using a Cavro XCalibur syringe pump (Tecan, Männedorf, Switzerland) and deposited the aliquots into individual wells of a microtiter plate sealed with a silicon seal to prevent evaporation using an XYZ positioning robot (Makeblock, Shenzhen, China). While depositing the aliquots into the microtiter plate, we used the syringe pump to mix the aliquots with glutaraldehyde (final concentration of 0.5% [vol:vol]), thereby fixing the cells and quenching further biological activity. We used these aliquots to quantify cell densities and extracellular nitrate (NO_3^-) and nitrite (NO_2^-) concentrations as described below.

We used flow cytometry to quantify cell densities during bioreactor operation. We first diluted the glutaraldehyde-fixed cells in 10 mM Tris EDTA (pH 8) buffer supplemented with SYBR Green I dye dissolved in dimethyl sulfoxide (ThermoFisher

Scientific, Waltham, MA). We incubated the cells with SYBR Green I for 30 minutes and then counted the number of SYBR Green I-stained cells with a Gallios (Beckman Coulter, Brea, CA) or an Accuri C6 (BD Biosciences, Eysins, Switzerland) flow cytometer. We separated SYBR Green I-stained cells from background signal as described elsewhere [2, 3].

We used ion chromatography (IC) to measure extracellular nitrate (NO_3^-) and nitrite (NO_2^-) concentrations during bioreactor operation. We first filtered the glutaraldehyde-fixed samples through 0.20 µm hydrophilic filters and diluted the filtrates 25-fold in deionized water. We determined extracellular nitrate and nitrite concentrations in the filtered and diluted samples using an IC Flex 930 chromatography system equipped with a Metrosep A Supp 16 - 250/4.0 column (Metrohm, Herisau, Switzerland) as we described elsewhere [4]. We used chloride as an internal reference.

Spiral plating and fluorescence photography. To quantify the ratios of the specialist to generalist ($r_{S/G}$s) of co-cultures, we used a spiral plating method in conjunction with fluorescence photography. This approach allowed us to obtain more than 2000 isolated colonies per LB agar plate, which was sufficient for us to accurately quantify $r_{S/G}$ at the lowest observed frequencies of the generalist or specialist and to distinguish the specialist from the generalist.

To perform spiral plating, we used a modified version of a method described elsewhere [5] and presented in Supplementary Fig. S3. We first diluted co-cultures in

0.85% sodium chloride (NaCl) solution by 200-fold. We then attached a fresh LB agar plate to the top of a mini lab-top centrifuge (500 rpm), dipped a sterile toothpick into the diluted co-cultures, and made a slow streak from the center to the rim of the rotating LB agar plate. This resulted in a dense spiral-shaped distribution of colonies on the LB agar plate (Supplementary Fig. S3D). To reduce autofluorescence from the LB agar and improve contrast between colonies and LB agar, we added 5 g of powdered activated carbon (Sigma-Aldrich, St. Louis, MO) per liter of LB agar prior to autoclaving.

To perform fluorescence photography, we exploited the fact that the specialist expresses either the *egfp* or *echerry* fluorescent protein-encoding gene while the generalist does not. We can therefore use fluorescence photography to distinguish colonies of the generalist and specialist. The fluorescence photography system consists of blue Oslon LD CQ7P LEDs with peak emission wavelengths at 455 nm (Osram, Münich, Germany), green Oslon LT CP7P LEDs with peak emission wavelengths at 528 nm, an EOS 1200D camera with an EF 50mm 1:1.8 STM lens (Canon, Tokyo, Japan), and a set of long-pass filters (Cokin, Paris, France). We captured photographs of *egfp*-expressing colonies by illuminating colonies with blue light and using the yellow long-pass light filter. Similarly, we captured photographs of *echerry*-expressing colonies by illuminating colonies with green light and using the red long-pass emission filter. Finally, we obtained reflected light photographs of all the colonies by illuminating them with green light and using (in this case needlessly – but for the sake of less handling) the yellow long-pass light filter. In the reflected light images,

colonies of the specialist and generalist were indistinguishable. See Supplementary Fig. S3A-S3C for an overview of the photography method.

We adjusted the exposure time as needed while keeping the lens aperture set at f/5.6 and sensor sensitivity at ISO 800. We obtained better images once we eliminated exposure to ambient light. We therefore kept the LEDs, LB agar plates and the camera in a black box shielded from ambient light (Supplementary Fig. S3A) and used tethering software for camera control and image acquisition (Canon EOS Utility and digiCamControl). Indirect and even (from all sides) LED illumination were critical for two reasons. First, when illuminated directly, colonies were recorded as two circular objects; one was the colony itself while the other was surface reflection. Surface reflection typically resulted in detecting additional (false positive) objects during automated colony calling. Second, the surface reflection intensity was much higher than the fluorescence signal and could therefore lead to false colony identification during automated analysis.

We analyzed the photographs in Matlab (MathWorks, MA, USA). After background removal (Supplementary Fig. S3D), round objects (in our case microbial colonies) were detected from the reflected light images with a circular Hough transform implemented with the 'imfindcircles' function. This allowed us to detect colonies without information of their fluorescence, thus avoiding analytical bias originating from the presence/absence of a fluorescence signal (Supplementary Fig. S3E). We took extra precautions to not to move the LB agar plate and the camera during imaging (both the camera and agar plate holder were surface mounted [Supplementary. Fig.

S3A]), which enabled us to retain spatial information – the colony center in the reflected light image therefore corresponds to the colony center in the fluorescence images. In the next step, we queried fluorescence images (Supplementary Fig. S3F) for fluorescence intensity in the colony center area (Supplementary Fig. S3G; red colonies expressed the *echerry* fluorescent protein-encoding gene). For example, if the captured fluorescent light intensity in an image exceeded the threshold for the area corresponding to the center of a colony, then this colony would be considered fluorescent. As we used only a small area around the colony center for this analysis (81 pixels in 3456 x 3456 pixel images), colony size had no effect on colony binning. If light intensity was below a threshold for both red and green fluorescent proteins, then it was considered as tag-free. In some cases, two merged colonies appeared as one single colony in the reflected light image (Supplementary Fig. S3G; bottom right corner). When of heterogenous origin, colonies would have high fluorescence signal variance in the image area used for colony identification, and were counted as double origin colonies. Finally, we calculated the likely number of undetected homogenous origin double colonies (double colonies with identical tag) from the frequency of detected double colonies and the frequency of each colony type, and corrected our colony counts for these values.

Delineating different individual-level phenotypes of the generalist. One explanation for the dynamics of the specialist is that the generalist diversifies its phenotype with regards to its nitrite (NO_2^-) utilization properties. Such diversification of the generalist could include phenotypes that occupy or deplete temporally available niche opportunities (*i.e.*, nitrite availability) for the specialist. To test for diversification in

nitrite utilization properties by the generalist, we isolated 420 individual colonies of the generalist from one evolved co-culture series (6 x 70 colonies) serially transferred at pH 6.5 (strong nitrite toxicity), and from four (out of eight) control generalist-only control cultures (4 x 70 colonies). We measured growth properties of those colonies in anoxic ACS medium containing nitrate (NO_3^-) as the growth limiting substrate and the pH set to 6.5 (strong nitrite toxicity). Briefly, we transferred each of the 70 colonies into a separate well of a single 96-well microtiter plate. As controls, we also transferred seven colonies of the ancestral generalist into wells of the same 96-well plate. We first archived the plate and then incubated it in anoxic conditions and collected OD_{600} measurements over time as described in the Materials and Methods section of the main text.

After completing the plate incubation and data acquisition, we first pooled all the recorded growth curves into one collection and identified distinct growth patterns using K-means clustering. We first performed K-means clustering along two properties; the duration of nitrite (NO_2^-). availability and the maximum observed OD_{600} at the end of growth with nitrate (NO_3^-). A subset of the generalist isolates did not show biphasic growth, and we could therefore not distinguish growth with nitrate from growth with nitrite. For these isolates, we set the maximum OD_{600} to that observed after the end of growth with both nitrate and nitrite. In total, this approach identified three main clusters based on the time lag between nitrate and nitrite reduction. One cluster had no time lag (Supplementary Fig. S8A; phenotype A), a second cluster had a short time lag (Supplementary Fig. S8B and S8D; phenotypes B

and D), and a third cluster had a long time lag (Supplementary Fig. S8C and S8E; phenotypes C and E).

After this first round of K-means clustering, we performed a second round of K-means clustering. In this second round, we excluded the cluster that had no observable time lag between nitrate (NO_3^-) and nitrite (NO_2^-) reduction (Supplementary Fig. S8A; phenotype A) from the dataset and separated the remaining isolates based on the difference in the OD_{600} at the beginning and end of the time lag period divided by the duration of the time lag period, and based on the ratio of the OD_{600} at the end of growth with nitrate and at the end of growth with both nitrate and nitrite. We performed this clustering because the OD_{600} was clearly lower at the end of the time lag period than at the beginning for some isolates (suggesting cell death, aggregation, or deposition to the edges of the microtiter plate wells), and the difference in the OD_{600} was proportional to the length of the time lag period. This clustering round effectively separated those phenotypes that did not show a reduction in OD_{600} during the time lag period (Supplementary Fig. S8B and S8C; phenotypes B and C) and those that did (Supplementary Fig. S8D and S8E; phenotypes D and E).

When we combined the results from both K-means clustering rounds, we distinguished four phenotypes. Phenotype B has a short time lag between nitrate (NO_3^-) and nitrite (NO_2^-) reduction and a consistent OD_{600} over that time (Supplementary Fig. S8B). Phenotype C has a long time lag and a consistent OD_{600} over that time (Supplementary Fig. S8C). Phenotype D has a short time lag and a decreasing

OD_{600} over that time (Supplementary Fig. S8D). Finally, phenotype E has a long lag phase and a decreasing OD_{600} over that time (Supplementary Fig. S8E).

Genome analyses. We sequenced the genomes of representative generalist isolates for each phenotype. Briefly, we streaked each generalist isolate onto LB agar plates, picked single colonies, and grew the generalist isolates overnight in oxic LB medium until reaching stationary phase. We then extracted and purified genomic DNA using the Wizard Genomic DNA purification kit (Promega, Madison, WI) and confirmed the quality of the DNA with gel electrophoresis. We then sent the purified genomic DNA to GATC Biotech AG (Konstanz, Germany) for sequencing with an Illumina HiSeq 4000 sequencer (Illumina, San Diego, CA). The sequencing was conducted with 150 cycles of paired-end sequencing and a target depth-of-coverage of 300-500x. Primary data and quality control analyses were performed using FastQC (Illumina, San Diego, CA). We reported the complete set of parameters used for quality control elsewhere [6, 7, 8].

We identified putative genetic differences between each generalist isolate serially transferred at pH 6.5 (strong nitrite [NO_2^-] toxicity) and its respective ancestor, including synonymous and non-synonymous mutations, insertions, deletions, and multiplications, in collaboration with the ETH Genetic Diversity Centre (Zürich, Switzerland). PRINSEQ-lite v0.20.4 was used to quality filter the raw reads, remove duplicate reads, and trim ambiguous base pairs [9]. breseq v.0.24rc5 was used to identify putative genetic differences relative to reference genomes [10]. The

procedures and parameters used for sequence analyses are identical to those reported in our previous studies [6, 7, 8].

Mathematical models and simulations. We used adapted versions of models formulated and described in detail elsewhere that simulate primary substrate use and intermediate substrate production and use by multiple cell-types growing within the same completely-mixed physical compartment [4, 11]. Growth of individual phenotypes are described by the Monod function. As cells consume the primary substrate, they produce a secondary substrate (metabolic intermediate) in an equimolar concentration to the consumed primary substrate (note, the reduction of one molar unit of nitrate [NO_3^-] produces one molar unit of nitrite [NO_2^-]). In our implementation of the model here, all phenotypes can consume and compete for the secondary substrate after it is produced.

We applied the model to simulate the invasion of the rare phenotypes (including evolved generalists) during serial transfer in completely mixed batch reactors, as we performed in our experiments. Thus, the final community composition at the end of one round of batch growth was the initial community composition in the subsequent round of batch growth. We adjusted the initial cell density of the subsequent round of batch growth to account for the appropriate dilution; in our case, we diluted the community 100-fold prior to inoculating into the subsequent round of batch growth. We solved the system of differential equations with the 'odeint' function in the SciPy package in Anaconda Python distribution (version 2020.02). The complete model is as follows:

Equations 1-3 below describe the growth of different phenotypes. Equation 1 describes the growth of the ancestral generalist, where $Cells_g$ is the cell concentration of the ancestral generalist, μ_{sg} is the specific growth rate of the ancestral generalist with substrate nitrate (NO_3^-), and μ_{ig} is the specific growth rate of the ancestral generalist with intermediate nitrite (NO_2^-). Similarly, Equation 2 describes the growth of the evolved generalist, where $Cells_e$ is the cell concentration of the evolved generalist, μ_{se} is the specific growth rate of the evolved generalist with NO_3^-, and μ_{ie} is the specific growth rate of the evolved generalist with NO_2^-. Equation 3 describes the growth of the specialist, where $Cells_s$ is the cell concentration of the specialist and μ_{is} is the specific growth rate of the specialist with intermediate nitrite (NO_2^-). Note that the specialist cannot use nitrate as a growth substrate.

Eq. 1. $$\frac{dCells_g}{dt} = Cells_g * \mu_{s_g} + Cells_g * \mu_{i_g}$$

Eq. 2. $$\frac{dCells_e}{dt} = Cells_e * \mu_{s_e} + Cells_e * \mu_{i_e}$$

Eq. 3. $$\frac{dCells_s}{dt} = Cells_s * \mu_{i_s}$$

Equations 4 and 5 below describe the depletion of the primary substrate and the production and depletion of the intermediate substrate. In our implementation, Equation 4 describes the depletion of nitrate (NO_3^-), where the terms $Yield_{sg}$ and $Yield_{se}$ are the yield coefficients for the ancestral and evolved generalist per unit of

nitrate consumed. Equation 5 describes the production and depletion of nitrite (NO_2^-), where $Yield_{ig}$ is the yield coefficient for the ancestral generalist growing with nitrite, $Yield_{ie}$ is the yield coefficient for the evolved generalist growing with nitrite, and $Yield_{is}$ is the yield coefficient for the specialist growing with nitrite.

Eq. 4. $$\frac{dSubstrate}{dt} = -(\frac{1}{Yield_{s_g}}) * Cells_g * μ_{s_g} - (\frac{1}{Yield_{s_e}}) * Cells_e * μ_{s_e}$$

Eq. 5. $$\frac{dIntermediate}{dt} = (\frac{1}{Yield_{s_g}}) * Cells_g * μ_{s_g} + (\frac{1}{Yield_{s_e}}) * Cells_e * μ_{s_e} -$$

$$(\frac{1}{Yield_{i_g}}) * Cells_g * μ_{i_g} - (\frac{1}{Yield_{i_e}}) * Cells_e * μ_{i_e} - (\frac{1}{Yield_{i_s}}) * Cells_s * μ_{i_s}$$

Equation 6 below describes the specific growth rate for the ancestral generalist growing with substrate nitrate (NO_3^-) and accounts for the inhibitory effects of intermediate nitrite (NO_2^-). Substrate is the concentration of nitrate, Intermediate is the concentration of nitrite, K_{m_sg} is the Monod coefficient for the ancestral generalist growing with nitrate, $K_{i\_Intermediate}$ is the inhibition coefficient for nitrite, and $μ_{sg\_max}$ is the maximal growth rate of the ancestral generalist with nitrate. The term $(\frac{Intermediate^2}{K_{i_{Intermediate}}})$ is the Andrews inhibition term that has been used previously to account for nitrite inhibition [4]. The term $(1 + \frac{Intermediate}{K_{mi_g}})$ describes the competitive inhibition of the nitrite intermediate and the nitrate substrate [12].

Eq. 6. $$μ_{s_g} = \frac{Substrate}{K_{ms_g} * (1 + \frac{Intermediate}{K_{mi_g}}) + Substrate + (\frac{Intermediate^2}{K_{i_{Intermediate}}})} * μ_{s_{g_{max}}}$$

Equation 7 below describes the specific growth rate of the ancestral generalist growing with nitrite (NO_2^-) and again accounts for the inhibitory effects of nitrite. K_{m_ig} is the Monod coefficient for the ancestral generalist growing with nitrite and μ_{ig_max} is the maximal growth rate of the ancestral generalist with nitrite. The term $(1 + \frac{Substrate}{K_{ms_g}})$ describes the competitive inhibition of the nitrite intermediate and the nitrate substrate.

Eq. 7.

$$\mu_{i_g} = \frac{Intermediate}{K_{m_{i_g}} * (1 + \frac{Substrate}{K_{ms_g}}) + Intermediate + (\frac{Intermediate^2}{K_{i_{Intermediate}}})} * \mu_{i_{gmax}}$$

Equation 8 below describes the specific growth rate of the specialist growing with nitrite (NO_2^-) and again accounts for the inhibitory effects of nitrite. K_{m_is} is the Monod coefficient for the specialist growing with nitrite and μ_{is_max} is the maximal growth rate of the specialist with nitrite. All other terms are identical to those presented in Equation 6.

Eq. 8.

$$\mu_{i_s} = \frac{Intermediate}{K_{m_{i_s}} + Intermediate + \frac{Intermediate^2}{K_{i_{Intermediate}}}} * \mu_{i_{smax}}$$

We could describe the growth of the evolved generalist by substantially increasing the term K_{m_se} (0.5) compared to the term K_{m_sg} (2×10^{-6}) in Equations 9 and 10. This approach simulates a scenario where the ability of the generalist to transport nitrate

(NO_3^-) from the bulk medium into the cell is impaired, thus reducing the accumulation of nitrite (NO_2^-) and preventing its toxic effects.

Eq. 9. $$\mu_{s_e} = \frac{Substrate}{K_{ms_e} * (1 + \frac{Intermediate}{Km_{i_e}}) + Substrate + (\frac{Intermediate^2}{K_{i_{Intermediate}}})} * \mu_{s_{e_{max}}}$$

Eq. 10. $$\mu_{i_e} = \frac{Intermediate}{K_{m_{i_e}} * (1 + \frac{Substrate}{Km_{s_e}}) + Intermediate + (\frac{Intermediate^2}{K_{i_{Intermediate}}})} * \mu_{i_{e_{max}}}$$

The parameters used for the simulations were estimated by comparing model outcomes with the OD_{600} data for selected isolates (Supplementary Fig. S4) and are as follows:

$\mu_{sg,max}$ = 0.47 (1/h)

$\mu_{ig,max}$ = 0.47 (1/h)

$\mu_{se,max}$ = 0.47 (1/h)

$\mu_{ie,max}$ = 0.47 (1/h)

$\mu_{is,max}$ = 0.47 (1/h)

$K_{m,sg}$ = 2×10^{-6} (mM)

$K_{m,ig}$ = 1 (mM)

$K_{m,se}$ = 0.5 (mM)

$K_{m,ie}$ = 1 (mM)

$K_{m,is}$ = 1 (mM)

$K_{i,Intermediate}$ = 6 (mM) for pH = 6.5, 40 (mM) for pH = 7.5

$Yield_{sg}$ = 0.01 (OD_{600}/mM substrate)

$Yield_{ig}$ = 0.0125 (OD_{600}/ mM intermediate)

$Yield_{se}$ = 0.01 (OD_{600}/mM substrate)

$Yield_{ie}$ = 0.0125 (OD_{600}/mM intermediate)

$Yield_{is}$ = 0.0125 (OD_{600}/mM intermediate)

Supplementary Results

Comparison of growth properties of individual phenotypes. Comparing the growth properties derived from the OD_{600} measurements for each of the five delineated phenotypes of the serially transferred generalist (Supplementary Fig. S8) reveals possible fitness advantages and tradeoffs. When grown in isolation, phenotype A has a higher maximum OD_{600} than any of the other phenotypes, but also a lower growth rate at low cell densities when nitrate (NO_3^-) is the predominant electron acceptor (Supplementary Figs. S4 and S8). However, it maintains a steady growth rate at intermediate cell densities when the other phenotypes enter the lag phase (Supplementary Figs. S4 and S8). It therefore may have a growth advantage during this period. Phenotype E, on the other hand, has the lowest maximum OD_{600} measurements but a higher OD_{600} when growing with nitrate and a higher growth rate when growing with nitrite (NO_2^-) (Supplementary Fig. S8). Of the four phenotypes with observed time lags between nitrate and nitrite reduction, phenotypes B and D spend less time in the lag phase. (Supplementary Fig. S8). They are second only to phenotype A with regards to the maximum OD_{600} and have the highest growth rates at low cell

densities (not significant for phenotype D). On the other hand, phenotype B has lower growth rates with nitrite than phenotypes D and E isolates. Overall, our data suggest many possible tradeoffs may exist between different growth properties with nitrate and nitrite.

Genome sequencing of individual-level phenotypes of the generalist. We sequenced whole genomes of several randomly chosen representatives of each phenotype (Supplementary Fig. S8). Of the 43 sequenced isolates (including several ancestral isolates), 19 have genetic differences when compared to their respective ancestor; 15 have one genetic difference, three have two genetic differences, and one has three genetic differences (Supplementary Table S2). All isolates belonging to phenotypes A, D, and E have at least one genetic difference, while only one isolate displaying phenotype B has a genetic difference. All isolates displaying phenotype D and phenotype E have genetic differences in genes associated with flagella/motility, either encoding structural proteins, or proteins involved in flagella regulation and folding (Supplementary Table S2).

Approximately 85% of the analyzed ancestral isolates display phenotypes classified as phenotype C, 15 % display phenotypes classified as phenotype B, and none display phenotypes classified as phenotypes A, D, or E (Supplementary Table S2). We sequenced six isolates that display phenotype B from serially transferred populations and three ancestral isolates that also display phenotype B. Aside from one isolate that displays phenotype B with a synonymous CAG>CAA mutation (Supplementary Table S2), we could not detect any genetic differences in that phenotype when compared to

the ancestors, or when compared to isolates that display the ancestral-like phenotype C. Similarly, some isolates display phenotypes D and E have insertion mutations in the same genes (*flgD* and *flgE*) (Supplementary Table S2).

We sequenced five isolates that display phenotype A from different co-cultures. Three have genetic changes in the gene encoding NarM (nitrate/nitrite transporter protein), of which one is a frameshift mutation and two introduce premature stop codons (Supplementary Table S2). One of these isolates has additional mutations in the cell division *ftsQ* gene and in a putative FAD-dependent oxidoreductase-encoding gene (Supplementary Table S2). A fourth isolate that displays phenotype A has a nonsynonymous mutation that replaces Met with Ile in the NarK nitrate/nitrite transporter, while the fifth isolate that displays phenotype A has a (TGCTGG)2→3 mutation in the gene encoding cytochrome B protein (Supplementary Table S2). These results suggest that phenotype A likely has a genetic basis.

Supplementary References

1 Coyle CL, Zumft WG, Kroneck PM, Korner H, Jakob W. Nitrous oxide reductase from denitrifying *Pseudomonas perfectomarina*. Purification and properties of a novel multicopper enzyme. Eur J Biochem. 1985;153:459–67.

2 Prest EI, Hammes F, Kötzsch S, van Loosdrecht MCM, Vrouwenvelder JS. Monitoring microbiological changes in drinking water systems using a fast and reproducible flow cytometric method. Water Res. 2013;47:7131-42.

3 Van Nevel S, Koetzsch S, Weilenmann HU, Boon N, Hammes F. Routine bacterial analysis with automated flow cytometry. J Microbiol Methods. 2013;94:73-6.

4 Lilja EE, Johnson DR. Segregating metabolic processes into different microbial cells accelerates the consumption of inhibitory substrates. ISME J. 2016;10:1568-78.

5 Gilchrist JE, Campbell JE, Donnelly CB, Peeler JT, Delaney JM. Spiral plate method for bacterial determination. Appl Environ Microbiol. 1973;25:244-52.

6 Lilja EE, Johnson DR. Metabolite toxicity determines the pace of molecular evolution within microbial populations. BMC Evol Biol. 2017;17:52.

7 Marchal M, Goldschmidt F, Derksen-Müller SN, Panke S, Ackermann M, Johnson DR. A passive mutualistic interaction promotes the evolution of spatial structure within microbial populations. BMC Evol Biol. 2017;17:106.

8 Lilja EE, Johnson DR. Substrate cross-feeding affects the speed and trajectory of molecular evolution within a synthetic microbial assemblage. BMC Evol Biol. 2019;19:129.

9 Schmieder R, Edwards R. Quality control and preprocessing of metagenomic datasets. Bioinformatics. 2011;27:863-4.

10 Deatherage DE, Barrick JE. Identification of mutations in laboratory evolved microbes from next-generation sequencing data using breseq. Methods Mol Biol. 2014;1151:165–8.

11 Dolinšek J, Goldschmidt F, Johnson DR. Synthetic microbial ecology and the dynamic interplay between microbial genotypes. FEMS Microbiol Rev. 2016;40:961-79.

12 Alvarez-Cohen L, McCarty PL. Product toxicity and cometabolic competitive inhibition modeling of chloroform and trichloroethylene transformation by methanotrophic resting cells. Appl Environ Microbiol. 1991;57:1031-7.
